# The Immunosuppressive Properties of Cyclo-[*D*-Pro-Pro-*β*^3^-HoPhe-Phe-] Tetrapeptide Selected from Stereochemical Variants of Cyclo-[Pro-Pro-*β*^3^-HoPhe-Phe-] Peptide

**DOI:** 10.3390/pharmaceutics16081106

**Published:** 2024-08-22

**Authors:** Krzysztof Kaczmarek, Jolanta Artym, Joanna Bojarska, Barbara Pacholczyk-Sienicka, Joanna Waśko, Ingrid Jelemenska, Wojciech M. Wolf, Martin Breza, Michał Zimecki

**Affiliations:** 1 Institute of Organic Chemistry, Łódź University of Technology, S. Żeromskiego Str. 116, 90-924 Łódź, Poland; barbara.pacholczyk@p.lodz.pl (B.P.-S.); joanna.wasko@p.lodz.pl (J.W.); 2 Department of Experimental Therapy, Hirszfeld Institute of Immunology and Experimental Therapy, R. Weigla Str. 12, 53-114 Wrocław, Poland; jolanta.artym@hirszfeld.pl (J.A.); michal.zimecki@hirszfeld.pl (M.Z.); 3 Institute of Inorganic and Ecological Chemistry, Chemistry Department, Łódź University of Technology, S. Żeromskiego Str. 116, 90-924 Łódź, Poland; wojciech.wolf@p.lodz.pl; 4 Department of Physical Chemistry, Slovak Technical University, Radlinskeho 9, SK-81237 Bratislava, Slovakia; ingrid.jelemenska@stuba.sk (I.J.); martin.breza@stuba.sk (M.B.)

**Keywords:** cyclic tetrapeptide, *D*-amino acids, contact sensitivity, carrageenan-induced inflammation, dextran sulfate-induced colitis, artificial intelligence, ADME-T profile

## Abstract

The anti-inflammatory, antiviral, and anti-cancer properties, as well as the mechanism of action of cyclo-[Pro-Pro-*β*^3^-HoPhe-Phe-] tetrapeptide (denoted as 4B8M), were recently described. The aim of this work was to synthesize and evaluate the immunosuppressive actions of the stereochemical variants of 4B8M by sequential substitution of *L*-amino acids by *D*-amino acids (a series of peptides denoted as P01–P07) using parent 4B8M as a reference compound. In addition, diverse available bioinformatics tools using machine learning and artificial intelligence were tested to find the bio-pharmacokinetic and polypharmacological attributes of analyzed stereomers. All peptides were non-toxic to human peripheral blood mononuclear cells (PBMCs) and only cyclo-[*D*-Pro-Pro-*β*^3^-HoPhe-Phe-] peptide (P03) was capable of inhibiting mitogen-induced PBMC proliferation. The peptides inhibited the lipopolysaccharide (LPS)-induced production of tumor necrosis factor-alpha (TNF-α) to various degrees, with P04 (cyclo-[Pro-Pro-*D*-*β*^3^-HoPhe-Phe-]) and P03 being the most potent. For further in vivo studies, P03 was selected because it had the combined properties of inhibiting cell proliferation and TNF-α production. P03 demonstrated a comparable ability to 4B8M in the inhibition of auricle edema and lymph node cell number and in the normalization of a distorted blood cell composition in contact sensitivity to the oxazolone mouse model. In the mouse model of carrageenan-induced inflammation of the air pouch, P03 exhibited a similar inhibition of the cell number in the air pouches as 4B8M, but its inhibitory effects on the percentage of neutrophils and eosinophils in the air pouches and blood, as well as on mastocyte degranulation in the air pouches, were stronger in comparison to 4B8M. Lastly, in a mouse model of dextran sulfate-induced colitis, similar effects to 4B8M regarding thymocyte number restoration and normalization of the blood cell pictures by P03 were observed. In summary, depending on either experimental findings or in silico predictions, P03 demonstrated comparable, or even better, anti-inflammatory and bio-pharmacokinetic properties to 4B8M and may be considered as a potential therapeutic. The possibility of P00 and P03 identification by circular dichroism measurements was tested by quantum-chemical calculations.

## 1. Introduction

Short peptides have attracted increasing attention in modern drug development due to their unique features, combining the advantages of small chemical molecules and biologics [1]. Thus, their function is difficult to mimic by traditional compounds. In addition, structural modifications, such as cyclization or incorporation of D-amino acids, as well as recent biotechnological progress, can overcome peptide limitations (mainly proteolytic degradation), leading to improved pharmacokinetic and pharmacodynamics profiles [1].

Notably, natural and synthetic cyclic peptides belong to promising immunosuppressive therapeutics [2,3]. Ultra-short cyclic peptides show enhanced bioavailability compared to linear and longer peptides, since they are resistant to proteolysis and exhibit increased cell membrane permeability [4,5]. 

Cyclolinopeptide A (CLA) from linseed oil was described as an inhibitor of several types of immune response, such as humoral and cellular immune response, skin graft rejection, and graft versus host reactions [6]. The potency of CLA in these experimental models was comparable to that of cyclosporine A (CsA). Although CLA was shown to bind to cyclophilin, similarly to CsA [7], the affinity of this interaction was weak [8], suggesting that another mechanism was responsible for its immunosuppressive activity.

Based on the structure of CLA (Pro-Pro-Phe-Phe-Leu-Ile-Ile-Leu-Val), numerous modifications of the peptide have been performed, including linear and cyclic variants, chain shortening, and amino acid substitutions, as well as establishing an essential four-amino acid sequence [9]. Eventually, a cyclic [Pro-Pro-*β*^3^-HoPhe-Phe-] tetrapeptide (denoted 4B8M) was synthesized, exhibiting anti-inflammatory properties in several in vivo mouse models [10,11]. In addition to revealing its mechanism of action [11], the antiviral properties [12] and antitumor properties [13] of the peptide have recently been described.

Although the effects of the substitution of cyclic cyclolinopeptides with unnatural amino acids on their immunosuppressive properties have been studied [14,15,16,17,18], no attempt has been made to investigate the consequences of the replacement of *L*-amino acids by *D*-amino acids in such peptides (containing Pro-Pro-Phe-Phe sequence), and particularly in the 4B8M cyclic tetrapeptide. Such modifications could lead to an improvement in immunosuppressive potency by the parent peptide and/or better bioavailability, as found in other studies [19,20].

In addition, we decided to check differences in the bio-pharmacokinetic parameters of stereomers by testing free available online web tools using diverse machine learning methods and artificial intelligence. The application of modern bioinformatics software is crucial for rapid, thorough, and precise prediction of the ADMET profile (adsorption, distribution, metabolism, excretion, and toxicity) and other bio-pharmacological properties before they undergo very expensive and time-consuming clinical trials [21]. It is well-known that the development of safe and effective therapeutic agents is a complex, time-consuming (takes more than ten years), and costly (more than USD 2.6 billion) process [22]. The failure of candidates in clinical trials is the main problem in drug research and development. Therefore, computational approaches may significantly improve the identification of lead molecules with high efficacy by the early detection of toxic and unfavorable side effects. They also enable insight into the structure–activity relationship, which makes the drug optimization process easier via the indication of modifications that improve potency and reduce toxicity. Machine learning methods integrated with artificial intelligence have become the indispensable tools of a new era of pharmacovigilance that can further decrease the cost of clinical trials by also improving the success rate. The biggest pharmaceutical companies collaborate with information technology companies to develop artificial intelligence-based methods for drug design and development. It can be mentioned that a new promising drug for idiopathic pulmonary fibrosis was discovered by in silico medicine aided by artificial intelligence (https://clinicaltrials.gov/ct2/show/NCT05154240, accessed on 1 July 2024) [23]. There is no doubt that artificial intelligence is revolutionizing the healthcare sector. Nevertheless, its true impact is yet to be fully realized [24].

It should be highlighted that popular free online servers do not always take into account structural nuances (such as absolute configuration) or are not reliable for larger structures, which we visualized in this paper.

Considering all these points in the course of our studies on the ultra-short cyclo-peptides, this study aimed to synthesize a series of peptides by the consecutive replacement of *L*-amino acids by *D*-amino acids in the 4B8M peptide (see Table 1) and evaluate how these modifications would affect their immunosuppressive properties in vitro, as compared to the parent 4B8M peptide. The peptide with the most promising activity will be selected for further in vivo studies in parallel with its parent 4B8M peptide. The additional aim of this study was to find a reasonable bio-pharmacokinetic profile for these stereomers through the critical examination of diverse machine learning methods supported by artificial intelligence. We also checked the polypharmacological properties of the analyzed molecules. Polypharmacology is an emerging strategy in the discovery and development of novel drugs. The complexity of diverse diseases calls attention to the relevance of multi-target therapeutic agents as effective drug candidates [25]. Using in silico tools to predict these aspects facilitates the process of drug development, reducing costs [26,27].

In our previous study [13], DFT studies of cyclo(Pro-Pro-*β*^3^homoPhe-Phe-), denoted as 4B8M, have been performed. Its optimized geometry, atomic charges, and relevant molecular orbitals in vacuum and aqueous solutions were described. These quantities barely change by replacing their L-amino acids with D-amino acids. Therefore, the problem of identifying the obtained peptides in this way arises. Fortunately, their optical activity can be utilized, and circular dichroism measurements can be used for this purpose. Quantum-chemical calculations are able to predict both the electronic (ECD) and vibrational (VCD) circular dichroism spectra of individual compounds. Our additional aim is to investigate the suitability of these spectral methods for the identification of the compounds under study. 

## 2. Materials and Methods

### 2.1. Reagents

Phosphate-buffered saline solution (PBS) and Hanks’ medium were derived from The Institute of Immunology and Experimental Therapy, Wrocław, Poland. The RPMI-1640 medium was from Cibi/Life Technologies, Gibco (Paisley, UK). Fetal calf serum (FCS) was from Gibco. Actinomycin D, dimethyl sulfoxide (DMSO), phytohemagglutinin A (PHA), lipopolysaccharide (LPS) from *Escherichia coli* strain O111:B4, 93-[4,5-dimethylthiazol-2-yl]-2,5-diphenyltetrazolium bromide (MTT), sodium dodecyl sulfate (SDS), *N*,*N*-dimethylformamide (DMF), 4-ethoxymethylene-2-phenyl-2-oxazolin-5-one (oxazolone), and carrageenan were from Sigma-Aldrich (Darmstadt, Germany) and dextran sulfate 40 KDa was from TdB Consultancy AB, Uppsala, Sweden. Mouse fibrosarcoma WEHI 164.13 cells (ATCC CRL 1751) were derived from the collection of cell lines at The Institute of Immunology and Experimental Therapy, Wroclaw, Poland. All reagents used in the UHPLC-MS analysis are certified LC-MS purity grade. 

All Fmoc-amino acids and D-Phe-2-ChloroTrityl and L-Phe-2-ChloroTrityl resins, as well as peptide synthesis reagents, were generous gifts from Chem-impex (Wooddale, IL, USA) and IRIS GmbH (Marktredwitz, Germany). All solvents used for automated SPPS were from Baker (USA). DCM and other solvents used for linear peptide cyclization and preparative purification cyclic ones used in HPLC were Fluka or Baker grade.

Peptide synthesis details and purification will be described in the Supplementary Materials file. The peptides were initially dissolved in DMSO (5 mg/mL) and subsequently in the culture medium to a desired concentration.

Chemical structures of the cyclic peptides are presented in Table 1.

It can be mentioned that two pairs of enantiomers, such as P00 (4B8M) and P06, as well as P02 and P04, represent stereochemical correlations. All others are diastereomers. 

We were planning the synthesis of all other stereoisomers of 4B8M (15 out of 16), which was labeled from this moment as P00 (means P01 to P15). We split all analogs into two groups, the first group containing stereomers with a single D-amino acid replacement and four D-amino acids (an enantiomer of 4B8M), and the next two with two and/or three D-amino acids picked up randomly. We found only one analog in the first group, P03, which is promising in view of its activity, so we postponed the laborious synthesis, cyclization, and purification of the other stereomers. We switched to the analogs containing a single replacement of one of two prolines and phenylalanine by coded amino acid residues (published elsewhere).

### 2.2. Isolation of Human Peripheral Blood Mononuclear Cells (PBMCs)

Venous blood was taken from a single donor into heparinized syringes and diluted twice with PBS. PBMCs were isolated by centrifugation on a Ficoll-uropoline gradient (density 1.077 g/mL) (Lymphoprep, PAA Laboratories, Linz, Austria), at 800× *g* for 20 min at 4 °C. The interphase cells were then washed three times with Hanks’ medium and re-suspended in the culture medium (RPMI-1640 with 10% addition of FCS, *L*-glutamine, sodium pyruvate, 2-mercaptoetanol, and the antibiotics streptomycin and penicillin) at a density of 2 × 10^6^ cells/mL. 

### 2.3. Proliferative Response of PBMC to PHA

The isolated PBMCs were distributed into 96-well flat-bottom plates in 100 µL aliquots (2 × 10^5^ cells/well). PHA was used at a concentration of 5 µg/mL. The compounds were tested at concentrations of 1, 10, and 100 µg/mL. DMSO at appropriate dilutions served as a control solvent. After a 4-day incubation in a cell culture incubator, the proliferative response of the cells was determined by the colorimetric MTT method [28] in a Dynatech 5000 spectrophotometer. Data (OPSYS MR Dynex Technologies, Chantilly, VA, USA) are presented as a mean optical density (OD) at 550 nm, with a reference wavelength of 630 nm, from quadruplicate wells ± standard error (SE). The control cultures contained no PHA or PHA without the studied compounds.

### 2.4. The Toxicity Test

PBMCs, at a density of 2 × 10^5^/100 µL/well and re-suspended in culture medium, were cultured for 24 h in a cell culture incubator with the compounds (1, 10, and 100 µg/mL concentrations). Cell survival was determined by the MTT colorimetric method [28]. Data are presented as a mean OD value from quadruplicate wells ±SE. ‘Control (-)’ cultures contained only cells in the culture medium.

### 2.5. Determination of TNF-α Activity Using a Bioassay

Human whole blood was diluted 10× with RPMI-1640 medium and distributed to 24-well culture plates in 1 mL aliquots. LPS was added to the culture at a concentration of 1 µg/mL. The studied peptides were used at concentrations of 1, 10, and 100 µg/mL. After overnight incubation, the supernatants were harvested and frozen at −20 °C until cytokine determination. TNF-α activity was determined using a bioassay with the highly sensitive cell line WEHI 164.13 [29]. Briefly, cells were seeded at a concentration of 2 × 10^4^ cells/well in quadruplicate. Increasing dilutions of the assayed supernatant were mixed with the target cells in the presence of actinomycin D (1 µg/mL). After 20 h of incubation, MTT was added into the wells, and the cultures were incubated for an additional 4 h. Next, a lysing buffer (20% SDS with 50% DMF, pH 4.7) was added and the OD at 550/630 nm in a spectrophotometer was measured after 24 h. The detection limit of the assay was about 2.5 pg/mL. One unit of TNF-α activity was defined as an inverse of supernatant dilution where 50% cell death took place. No statistics were applied here since the data were derived from single cultures (wells).

### 2.6. Colorimetric MTT Assay for Cell Growth and Death 

The assay was performed by the MTT colorimetric method [29]. Briefly, 25 µL of MTT (5 mg/mL) stock solution was added per well at the end of cell incubation and plates were incubated for 3 h in a cell culture incubator. Then, 100 µL of the lysing buffer (20% SDS with 50% DMF, pH 4.7) was added. After additional overnight incubation, the OD was measured at 550/630 nm (Dynatech 5000 spectrophotometer). 

### 2.7. Mice

C57Bl/6 female mice, 8–10 weeks old and weighing 19–22 g, were delivered by the Breeding Centre of Laboratory Animals at the Institute of Occupational Medicine, Łódź, Poland and used for this study. Mice had free access to granulated food and filtered water. The local ethics committee at the Institute of Immunology and Experimental Therapy, Wrocław, Poland, approved this study (permission #37/2012).

### 2.8. Isolation of Thymocytes

The thymocytes were isolated by pressing the thymuses through plastic screens into cold PBS. After 2× wash with PBS, the cells were re-suspended in cold PBS containing 0.2% trypan blue. The viable cells were counted in a Bürker hemocytometer.

### 2.9. Analysis of the Peripheral Blood Picture

Blood smears were made on microscopic glasses, and the preparations were dried and stained with Giemsa and May–Grünwald dyes. The blood picture was evaluated in a light microscope at 1000× magnification in immersion oil. Up to 100 cells were counted per preparation. The results were shown as a percentage of mean values of respective cell types: mature neutrophils, band forms of neutrophils, eosinophils, lymphocytes, and monocytes. 

### 2.10. Contact Sensitivity to Oxazolone 

The test was performed as described by others [30], with some modifications. Mice were shaved on the abdomen and after 24 h, 100 µL of 0.5% oxazolone in acetone was applied topically. The peptides were applied in an ointment (vaseline and lanolin, 50/50 *w*/*w*) at a concentration of 0.1%, topically, on both sides of the ears. The contact sensitivity reaction was elicited 5 days later by applying 50 µL of 1% oxazolone in acetone on both sides of the ears. The peptides were applied 2 h after elicitation of the contact sensitivity. The ear edema was measured after 24 h using a spring caliper. The results were presented as an antigen-specific increase in ear thickness, i.e., the ear thickness of background (BG) mice (given only the eliciting dose of the antigen) was subtracted.

### 2.11. Carrageenan-Induced Inflammation of Air Pouches

Air pouches were formed by a subdermal injection into the dorsal area (under halothane anesthesia) of 5 mL of air (23 G × 11/4 needle, 5 mL syringe) [31]. On the next day, the air pouches were supplemented by 1 mL of air and injected with 0.5 mL of 1% carrageenan solution in PBS. The peptides were dissolved in DMSO (1 mg/100 µL DMSO) and supplemented with 0.9% NaCl solution to a volume of 0.2 mL. P03 and 4B8M peptides were injected into air pouches at a dose of 100 µg/mouse 30 min after carrageenan administration. BG mice received the respective amount of DMSO in 0.9% NaCl. Control mice were given only carrageenan solution plus the respective amount of DMSO. The next day, the mice were bled to perform blood smears and sacrificed. A total of 1 mL of 0.9% NaCl was injected into air pouches and the content aspirated. From this source, a total number was determined, the rest were centrifuged, and from a cell pellet, the smears were performed on microscopic glasses and stained with Giemsa and May Grunwald reagents. The cell composition of air pouch exudates was analyzed and the results were shown as a percentage (mean values of the respective cell types: neutrophils, band forms of neutrophils, eosinophils, basophils, lymphocytes, macrophages, and mastocytes).

### 2.12. Dextran Sulfate-Induced Colitis 

The mice drank acidified water (pH 3.05) containing 3.5% dextran sulfate for 6 days [32]. Then, until day 12 of the experiment, the mice drank water. The P03 and 4B8M peptides were initially dissolved in DMSO and subsequently in 0.9% NaCl and given to mice per os (by a gastric tube) at a daily dose of 100 µg on days 1–6 of the experiment. On day 12, the mice were sacrificed and the peripheral blood and thymuses were isolated for analysis. The mice from the background (BG) group only drank acidified water (without dextran sulfate) for 6 days and were given the solvent (DMSO) per os (by a gastric tube) on days 1–6 of the experiment. Control mice drank acidified water plus 3.5% dextran sulfate and were given only the solvent (DMSO) by gastric tube.

### 2.13. Statistics

Five to nine mice per group were used in the experiments. Results (where applicable) are presented as mean values ± standard error (SE). The Brown–Forsyth test was used to determine the homogeneity of variance between groups. When the variance was homogenous, analysis of variance (one-way ANOVA) was applied, followed by post hoc comparisons with Tukey’s test to estimate the significance of the differences between groups. Nonparametric data were evaluated with the Kruskal–Wallis analysis of variance (ANOVA of K–W), as indicated in the text. Significance was determined at *p* < 0.05. Statistical analysis was performed using STATISTICA 7.0 for Windows.

### 2.14. In Silico Studies

**ADME-T profile:** The drug-likeness and pharmacokinetics of the stereomers were evaluated with SwissADME [33] (http://www.swissadme.ch/index.php, accessed on 1 July 2024), pkCSM [34] (http://biosig.unimelb.edu.au/pkcsm, accessed on 1 July 2024), and ADMET-AI (ADMEAbsorption, Distribution, Metabolism, Excretion, and Toxicity-Artificial Intelligence) [35]. In addition, the toxicity effects were calculated by Pro-Tox II [36] and Osiris [37].

SwissADME (is the most popular free web software that enables fast prediction of drug-likeness and physicochemical features. It also provides a BOILED-egg model and bioavailability radar [33,38]. This information is suitable for further experimental studies in the development process of novel drugs.

**Bioactivity (drug) score prediction:** The drug score, predicted using the Molinspiration web-based tool (www.molinspiration.com, accessed on 1 July 2024), characterizes the general potential of a synthesized molecule to be a drug candidate in terms of regular human receptors such as GPCRs, ion channels, kinases, nuclear receptors, proteases, and enzymes [39].

**Determination of bioactivities:** Biological activity prediction was performed using PASS online version 2.0 [40] (http://www.way2drug.com/passonline/, accessed on 1 July 2024). The results provide predicted activities and the corresponding probabilities as Pa (to be active) and Pi (to be inactive), in a range from 0 to 1. The prediction is based on the structural formula of a molecule for more than 4000 types of bioactivities (with an average accuracy of >95%). The structure–activity relationship is considered in the training set, assuming >300,000 organic compounds [40]. Activity is probable when the Pa > Pi. A Pa > 0.7 means high predicted activity, while a Pa < 0.5 signifies low activity, but also a chance of finding a structurally new compound [41,42].

**Target prediction:** SwissTarget Prediction is used as in silico target fishing to predict potential targets by mining chemical databases to search for similarities. This idea is related to comparing the structure of a new compound with chemically similar known molecules that can interact with similar protein targets [43].

Simplified Molecular Input Line Entry Specifications (SMILEs), an ASCII string needed for the predictions, were generated in BIOVIA Draw [44] to represent the chemical structure of the compounds according to the structure of each molecule.

### 2.15. UHPLC-MS Analysis

The purity and structure of the selected stereoisomers were confirmed using a UHPLC Vanquish Flex chromatograph equipped with an Intensity Solo 2 C18 column from Bruker^®^, 100 × 2.1 mm, with pore size 100 Å, a thermostat at 30 °C, and a diode array detector (DAD) from Thermo Scientific (Waltham, MA, USA). The UHPLC system was coupled with an Impact II mass spectrometer from Bruker^®^ equipped with a VIP-HESI ionization source and a time-of-flight analyzer. The UHPLC parameters were as follows: gradient elution, component A—water, component B—acetonitrile, both with the addition of 0.1% FA; analysis program (B/A): 0–1 min 5/95, 1–10 min 50/50, 10–15 min 80/20, 15–17 min 80/20, 17–19 min 5/95, 19–20 min 5/95; eluent flow 0.3 mL/min; analytical wavelengths of the DAD detector: 214, 220, and 254 nm; injection volume 2 µL. The MS spectrometer parameters were as follows: drying gas flow—9.0 L/min; nebulizer temperature 220 °C and pressure 3.0 Bar; capillary voltage 4500 V; end plate offset voltage: −500 V; analyzed ion range 100–1000 (*m*/*z*). The recorded chromatograms were elaborated using DataAnalysis ver. 5.0 software from Bruker^®^.

**Sample preparation:** The L- (P00) and D-stereoisomers (P03) were dissolved in an aqueous solution of 0.1 L309. The external calibration of the MS instrument was performed using a 10 mM sodium formate solution. The registered UHPLC-MS spectra for L- and D-stereoisomers are presented in the Supplementary Materials (Figure S1).

### 2.16. NMR Spectroscopy

Measurements were performed on a Bruker Avance II Plus 16.4 T spectrometer (Bruker BioSpin, Rheinstetten, Germany). The operating frequencies were 700 and 175 MHz for the ^1^H and ^13^C experiments, respectively. The instrument was equipped with a 5 mm Z-gradient broadband decoupling inverse probe. The precise assignment of the ^1^H and ^13^C chemical shifts was performed by 2D NMR experiments. All chemical shifts were referenced to the DMSO-d6 signal at 2.48 ppm. 

For ^1^H spectra, 128 scans were acquired with a relaxation delay of 1s and other parameters set as follows: AQ = 2.27 s, TD = 64 k, SW = 20.60 ppm. Homonuclear correlated spectra (COSY) were acquired using a standard pulse sequence (cosygpqf). Spectra were recorded with the acquisition of 32 transients for each of the 256 increments with 2K data points. The spectral widths for 1H were 6313.13 Hz. Two-dimensional ^1^H-^13^C HSQC spectra were recorded using the Bruker pulse sequence—hsqcetgpsi2. The spectral widths for ^1^H and ^13^C were 6313.13 Hz and 31,706 Hz sampled with 2048 and 256 complex points, respectively. The number of scans was 32. Two-dimensional ^1^H-^13^C HMBC spectra were recorded using the Bruker pulse sequence—hmbcgplpndqf. The spectral widths for ^1^H and ^13^C were 6313.13 Hz and 40,481.75 Hz sampled with 2048 and 256 complex points, respectively.

### 2.17. Quantum-Chemical Calculations

We started the geometry optimizations of the neutral P03 molecule in the ground singlet spin state by a suitable modification of the 4B8M structure (P00) obtained in our previous study [13] using the Gaussian 09 program [45]. For this purpose, the density functional theory [46,47,48] with the hybrid functional B3LYP [49,50,51] and the 6–311++G(d,p) basis sets from the Gaussian library [45] were used. The Conductor-like Polarizable Continuum Model (CPCM) [52,53,54] was used to evaluate the effect of the aqueous environment. The optimized geometry was checked on the absence of imaginary vibrations by vibrational analysis. Infrared (IR) intensities and vibrational circular dichroism (VCD) rotational strengths were obtained using the keyword Freq = VCD [55].

Excitation energies, oscillator strengths, and electronic circular dichroism (ECD) rotatory strengths in a velocity gauge [56,57] were obtained within a time-dependent DFT (TD-DFT) method [58,59]. The VCD and ECD spectra were obtained by Gaussian widening of discrete lines with full widths at half maximum (FWHM) of 0.01 and 0.1 eV [60,61,62], with intensities related to the maximal peak.

The superposition of the optimized structures was optimized using the PyMol software, Ver. 1.8 [63].

## 3. Results

### 3.1. Estimation of the Purity of the D- and L-Stereoisomers

For both stereoisomers (P00 and P03), the calculated monoisotopic mass is equal to 502.2579 and the recorded MS spectra confirmed their structure (Figure S1a,c). For the L-stereoisomer (P00), the pseudomolecular ion [M+H]^+^ = 503.2667. In the case of the D-stereoisomer (P03), a peak [M+H]^+^ = 503.2652 was detected. Based on the HPLC chromatograms recorded at 214 nm (Figure S1b,d), the purity of the synthesized L and D stereoisomers was estimated to be similar, above 98%. It should be noted that retention times for both cyclopeptides were different, Rt = 10 min and Rt = 11.3 min for P00 and P03, respectively. This is a typical observation that allows one to distinguish the peptide sequences depending on the content of L- and D-amino acids.

### 3.2. Evaluation of Activities of the Peptides In Vitro

**Figure 1:**
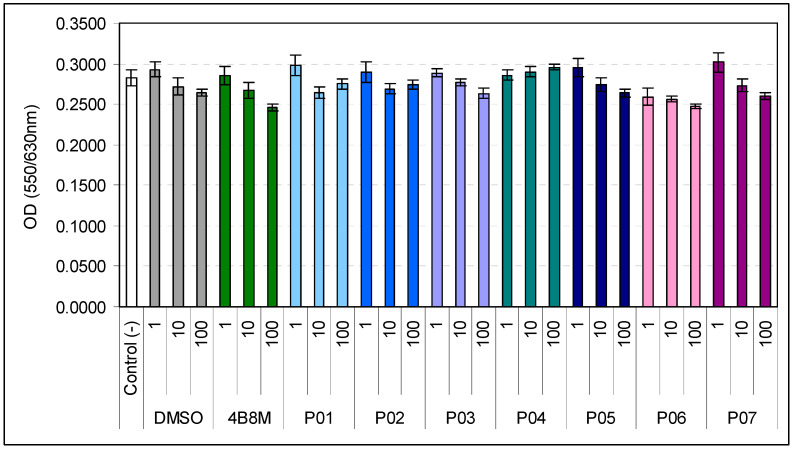
Effects of P01–P07 peptides on the viability of human PBMCs. Peptides P01–P07 and the reference compound 4B8M were tested at concentrations of 1, 10, and 100 µg/mL. The cell culture without DMSO and peptides is denoted as Control (-). Statistics (all comparisons vs. DMSO at appropriate dilutions): 1 µg/mL: 4B8M NS (*p* = 1.0000); P01 NS (*p* = 1.0000); P02 NS (*p* = 1.0000); P03 NS (*p* = 1.0000); P04 NS (*p* = 1.0000); P05 NS (*p* = 1.0000); P06 NS (*p* = 0.8253); P07 NS (*p* = 1.0000) (ANOVA); 10 µg/mL: 4B8M NS (*p* = 1.0000); P01 NS (*p* = 1.0000); P02 NS (*p* = 1.0000); P03 NS (*p* = 1.0000); P04 NS (*p* = 0.9999); P05 NS (*p* = 1.0000); P06 NS (*p* = 1.0000); P07 NS (*p* = 1.0000) (ANOVA); 100 µg/mL: 4B8M NS (*p* = 0.9999); P01 NS (*p* = 1.0000); P02 NS (*p* = 1.0000); P03 NS (*p* = 1.0000); P04 NS (*p* = 0.9047); P05 NS (*p* = 1.0000); P06 NS (*p* = 0.9999); P07 NS (*p* = 1.0000) (ANOVA).

The preliminary evaluation of the activity of the cyclic tetrapeptides included their effects on the PHA-induced proliferation of human PBMCs and the LPS-induced production of TNF-α by human whole blood cell cultures in the 1–100 µg/mL concentration range. All compounds were also tested for cell toxicity in the 1–100 µg/mL concentration range against human PBMCs. The effects of the peptides on PBMC survival in 24 h culture are presented in Figure 1. The 4B8M peptide was included as a reference compound. Appropriate dilutions of DMSO were added to the control cultures. The results showed no signs of toxicity of the compounds in the studied concentration range. 

**Figure 2:**
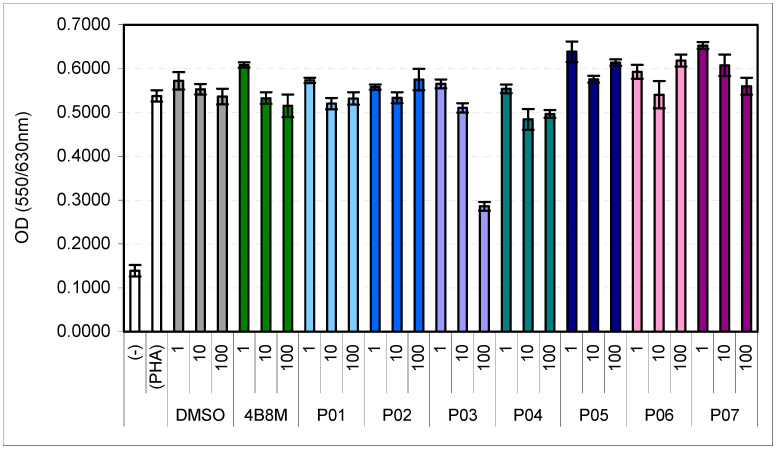
Effects of P01–P07 peptides on PHA-induced human PBMC proliferation. Peptides P01–P07 and the reference compound 4B8M were tested at concentrations of 1, 10, and 100 µg/mL. The cell culture without PHA, DMSO, and peptides is denoted as (-), and without DMSO and peptides as (PHA). Statistics (all comparisons vs. DMSO at appropriate dilutions): 1 µg/mL: 4B8M NS (*p* = 0.9995); P01 NS (*p* = 1.0000); P02 NS (*p* = 1.0000); P03 NS (*p* = 1.0000); P04 NS (*p* = 0.9047); P05 NS (*p* = 0.5198); P06 NS (*p* = 1.0000); P07 NS (*p* = 0.1445) (ANOVA); 10 µg/mL: 4B8M NS (*p* = 1.0000); P01 NS (*p* = 0.9999); P02 NS (*p* = 1.0000); P03 NS (*p* = 0.9930); P04 NS (*p* = 0.4297); P05 NS (*p* = 1.0000); P06 NS (*p* = 1.0000); P07 NS (*p* = 0.8647) (ANOVA); 100 µg/mL: 4B8M NS (*p* = 1.0000); P01 NS (*p* = 1.0000); P02 NS (*p* = 0.9982); P03 *p* = 0.0001; P04 NS (*p* = 0.9970); P05 NS (*p* = 0.2037); P06 NS (*p* = 0.1257); P07 NS (*p* = 1.0000) (ANOVA).

The effects of the peptides on the PHA-induced proliferative response of human PBMCs are presented in Figure 2. The 4B8M peptide was included as a reference compound. Appropriate dilutions of DMSO were added to the control cultures. The peptides are virtually devoid of anti-proliferative action except compound P03 at a concentration of 100 µg/mL. 

The effects of the peptides on LPS-induced TNF-α production in human whole blood cell cultures are presented in Table 2. The 4B8M peptide was included as a reference compound. Appropriate dilutions of DMSO were added to the control cultures.

The peptides displayed differential patterns of inhibition of LPS-induced TNF-α production. P03 and P04 demonstrated the best inhibition of TNF-α production with a moderate inhibition already registered at 1 µg/mL. Some of the compounds, including 4B8M, were weakly inhibitory, showing no dose-dependent inhibition or no inhibitory at all in the case of P06. Although the parent 4B8M did not show any significant effects on cell proliferation and TNF-α production in this set of experiments, other data (unpublished) revealed moderate inhibition of concanavalin A (ConA)-induced proliferation of mouse splenocytes.

The structure–activity relationships of the studied peptides are represented in Table 3. Most potent suppressive properties in the inhibition of LPS-induced TNF-α production were exhibited by P03 and P04, containing single *D*-Pro or *D*-*β*^3^-HoPhe in their structures, respectively. However, only P03, containing *D*-Pro, was also able to inhibit mitogen-induced cell proliferation at high concentrations. The ability to moderately inhibit TNF-α production was also preserved by P05, which also contains *D*-Pro, although in another position. P06, consisting exclusively of *D* amino acids, was completely inactive in both tests.

### 3.3. Contact Sensitivity to Oxazolone

**Figure 3:**
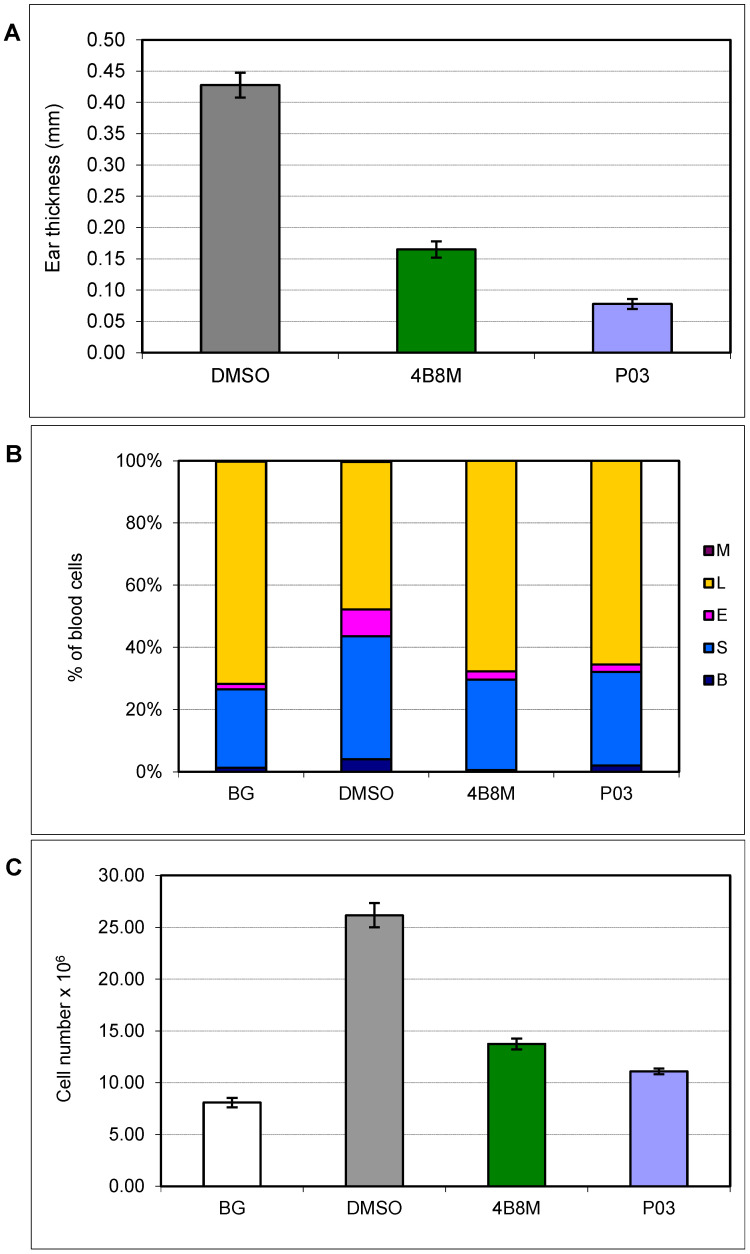
Effect of the P03 peptide on antigen-specific increase of ear edema (**A**), peripheral blood composition (**B**) and draining lymph node cell number (**C**) in a mouse model of contact sensitivity to oxazolone. P03 and 4B8M (as a reference compound) were applied to auricles in 0.1% ointment. BG indicates the control group that received only the eliciting dose of antigen to the ears (mice not sensitized). DMSO indicated the control group which was treated only with ointment containing DMSO. (**A**) The mean values ± SE of the antigen-specific increase of ear edema are presented in mm. Statistics: DMSO vs. 4B8M *p* = 0.0001; DMSO vs. P03 *p* = 0.0001; 4B8M vs. P03 *p* = 0.0003 (ANOVA). (**B**) The mean values of the percentage of blood cells are presented. B—bands (precursor forms of neutrophils), S—segments (mature forms of neutrophils), E—eosinophils, L—lymphocytes, M—monocytes. Statistics: B: DMSO vs. 4B8M *p* = 0.0017; DMSO vs. P03 NS (*p* = 0.1306) (ANOVA); S: DMSO vs. 4B8M *p* = 0.0097; DMSO vs. P03 NS (*p* = 0.0313) (ANOVA); E: DMSO vs. 4B8M *p* = 0.001; DMSO vs. P03 *p* = 0.0001 (ANOVA); L: DMSO vs. 4B8M *p* = 0.0001; DMSO vs. P03 *p* = 0.0001 (ANOVA). (**C**) The mean values ± SE of the number of cells in the draining lymph nodes are presented. Statistics: DMSO vs. 4B8M *p* = 0.0001; DMSO vs. P03 *p* = 0.0001 (ANOVA).

P03 in 0.1% ointment, applied 30 min after elicitation of contact sensitivity (CS) to oxazolone on auricles, was used to evaluate its suppressive effect on the effectual phase of CS (Figure 3A–C). The results show that an antigen-specific increase in ear edema was deeply suppressed by P03, even more strongly than by the parent, 4B8M (Figure 3A).

Local inflammatory responses, particularly if intense and involving large skin areas as in the case of CS, elicit generalized inflammation as reflected by the rapid release of neutrophils from the bone marrow. This is especially characteristic of rodents, which possess a big reservoir of neutrophils in the bone marrow [64]. As presented in Figure 3B, this is illustrated by a high increase in the percentage of myeloid cells (neutrophils and their precursors and eosinophils) in circulating blood. P03 significantly reduced the percentage of all myeloid cell types, to a similar degree as 4B8M.

The local inflammatory inflammation is typically reflected by a strong increase in the number of draining lymph node cells (Figure 3C). In addition, in the case of this parameter, P03 demonstrated a potent capability to lower the number of cells, with a similar or even higher degree as 4B8M.

### 3.4. Carrageenan-Induced Inflammation in Air Pouches

**Figure 4:**
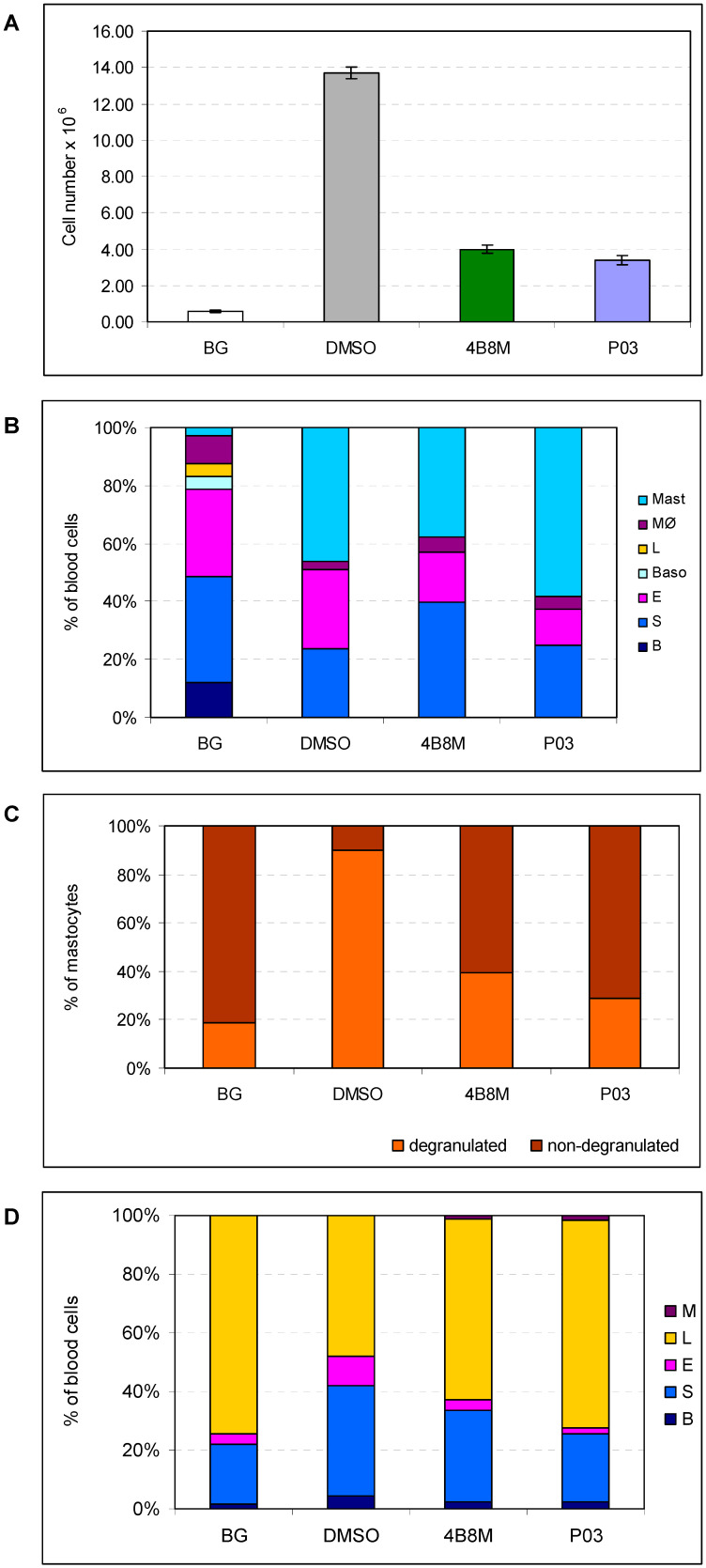
Effect of P03 peptide on air pouch exudate cell numbers (**A**), cell composition in air pouch exudates (**B**), mastocyte degranulation in air pouch exudates (**C**), and cell composition in peripheral blood (**D**) in a mouse model of carrageenan-induced nonspecific inflammation. P03 and 4B8M (as a reference compound) were injected into the air pouch at a dose of 100 µg/mouse, 30 min after carrageenan administration. BG indicates the control group that received, into the air pouch, the respective amount of DMSO in 0.9% NaCl (without previous application of carrageenan). DMSO indicates the control mice that received, into the air pouch, carrageenan solution plus the respective amount of DMSO. (**A**) The mean values ± SE of the number of peripheral blood cells are presented. Statistics: DMSO vs. 4B8M *p* = 0.0001; DMSO vs. P03 *p* = 0.0001 (ANOVA). (**B**) The mean values of the percentage of cells in the air pouch exudates are presented. B—bands (precursor forms of neutrophils), S—segments (mature forms of neutrophils), E—eosinophils, Baso—basophils, L—lymphocytes, MØ—macrophages, Mast—mastocytes. Statistics: B: DMSO vs. 4B8M NS (*p* = 0.1067); DMSO vs. P03 NS (*p* = 2771) (ANOVA of K-W); S: DMSO vs. 4B8M *p* = 0.0295; DMSO vs. P03 NS (*p* = 0.9997) (ANOVA): E: DMSO vs. 4B8M *p* = 0.0071; DMSO vs. P03 *p* = 0.0001 (ANOVA); Baso: DMSO vs. 4B8M NS (*p* = 1.0000); DMSO vs. P03 NS (*p* = 1.0000) (ANOVA of K-W); L: DMSO vs. 4B8M NS (*p* = 1.0000); DMSO vs. P03 NS (*p* = 1.0000) (ANOVA); MØ: DMSO vs. 4B8M NS (*p* = 0.1082); DMSO vs. P03 NS (*p* = 0.5997) (ANOVA); Mast: DMSO vs. 4B8M NS (*p* = 0.5849); DMSO vs. P03 NS (*p* = 0.1037) (ANOVA). (**C**) The mean values of the percentage of degranulated and non-degranulated mast cells in air pouch exudates are presented. Statistics: degranulated: DMSO vs. 4B8M *p* = 0.0001; DMSO vs. P03 *p* = 0.0001 (ANOVA); non-degranulated: DMSO vs. 4B8M *p* = 0.0001; DMSO vs. P03 *p* = 0.0001 (ANOVA). (**D**) The mean values of the percentage of blood cells are presented. B—bands (precursor forms of neutrophils), S—segments (mature forms of neutrophils), E—eosinophils, L—lymphocytes, M—monocytes. Statistics: B: DMSO vs. 4B8M NS (*p* = 0.1067), DMSO vs. P03 NS (*p* = 0.2771) (ANOVA); S: DMSO vs. 4B8M NS (*p* = 1.0000), DMSO vs. P03 *p* = 0.0172 (ANOVA of K-W); E: DMSO vs. 4B8M NS (*p* = 0.1178), DMSO vs. P03 *p* = 0.0007 (ANOVA of K-W); L: DMSO vs. 4B8M *p* = 0.0002, DMSO vs. P03 *p* = 0.0001; M: DMSO vs. 4B8M *p* = 0.0084, DMSO vs. P03 *p* = 0.0001 (ANOVA).

Carrageenan-induced inflammation is a non-antigen-specific mouse model reflecting clinical situations in rheumatoid arthritis [31]. P03 peptide injected into an air pouch at a dose of 100 µg/mouse, 30 min after carrageenan administration, was used to evaluate its suppressive effect on the development of the inflammatory response (Figure 4).

The level of exudate cells in air pouches, consisting mainly of neutrophils, eosinophils, and mastocytes, is strongly elevated (Figure 4A). The studied peptide P03 and the control 4B8M peptide, administered to air pouches, significantly decreased cell numbers to a similar degree. 

It also appeared that the compounds did not only lower the total cell number in the exudates but also changed the cell type composition (Figure 4B). These changes were more evident in the case of P03, where the content of eosinophils decreased with a concomitant increase in mastocytes.

More pronounced effects of P03 were observed when the percentages of degranulated and intact mastocytes in air pouches were analyzed (Figure 4C). Control, untreated air pouches contained 89.7%, 4B8M-treated air pouches contained 39.5%, and P03-treated air pouches contained 28.7% of degranulated mastocytes.

Lastly, P03 treatment appeared to be more effective than 4B8M in normalizing a distorted blood cell type composition in peripheral blood (Figure 4D). In particular, the percentages of neutrophils and eosinophils were lower. The blood of control mice consisted of 37% neutrophils and 9.6% eosinophils. Treatment of mice with 4B8M lowered the levels of the respective cell types to 31.5% and 3.4%, and treatment with P03 led to levels of 23% and 2.0%. 

### 3.5. Effect of P03 on Selected Parameters in Dextran Sulfate-Induced Colitis

**Figure 5:**
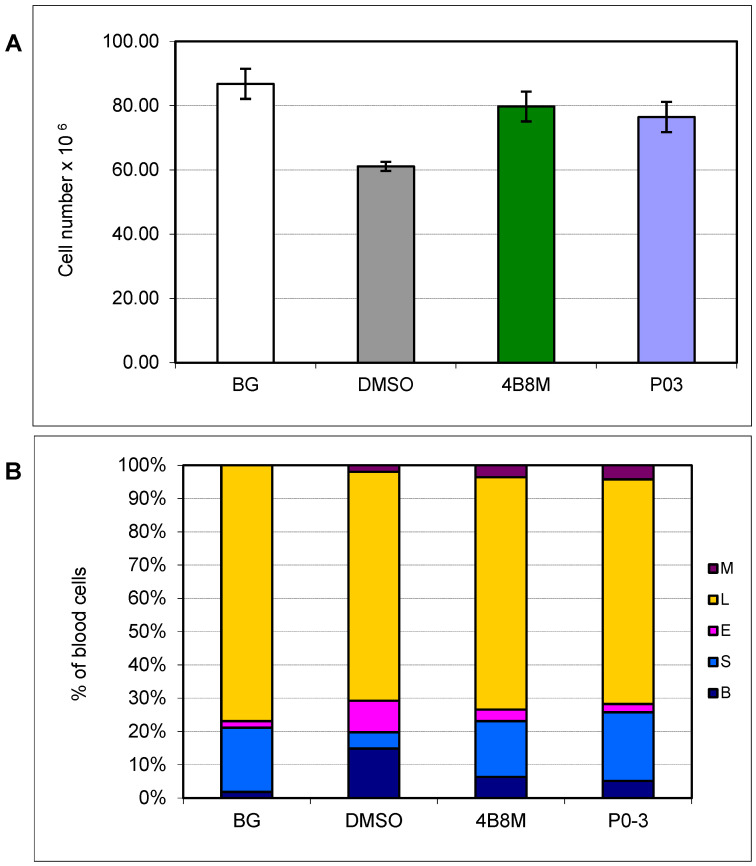
Effect of the P03 peptide on the number (**A**) and cell composition in peripheral blood (**B**) in a mouse model of dextran sulfate-induced nonspecific inflammation. P03 and 4B8M (as a reference compound) were applied to mice by gastric tube at a dose of 100 µg/mouse. The mice from the BG group only drank acidified water (without dextran sulfate) and were given the solvent (DMSO) per os (by a gastric tube). Control mice drank acidified water plus 3.5% dextran sulfate and were given only the solvent (DMSO) by gastric tube. (**A**) The mean values ± SE of the number of thymocytes are presented. Statistics: DMSO vs. 4B8M *p* = 0.0143, DMSO vs. P03 *p* = 0.0382 (ANOVA). (**B**) The mean values of the percentage of blood cells are presented. B—bands (precursor forms of neutrophils), S—segments (mature forms of neutrophils), E—eosinophils, L—lymphocytes, M—monocytes. Statistics: B: DMSO vs. 4B8M NS (*p* = 0.3828), DMSO vs. P03 *p* = 0.0111 (ANOVA of K-W); S: DMSO vs. 4B8M *p* = 0.0001, DMSO vs. P03 *p* = 0.0001 (ANOVA); E: DMSO vs. 4B8M NS (*p* = 0.2381), DMSO vs. P03 *p* = 0.0007 (ANOVA of K-W); L: DMSO vs. 4B8M NS (*p* = 0.9657), DMSO vs. P03 NS (*p* = 0.9927) (ANOVA); M: DMSO vs. 4B8M NS (*p* = 0.5438), DMSO vs. P03 NS (*p* = 0.0601) (ANOVA of K-W).

Dextran sulfate-induced nonspecific inflammation of the colon represents another type of physiological insult and is a model for the clinical equivalent of irritable bowel disease [32]. The P03 peptide and the 4B8M peptide (as the control) were applied by gastric tube at a dose of 100 µg/mouse to evaluate their suppressive effects on the development of the inflammatory response in the colon (Figure 5A).

Stressful conditions cause stimulation of endogenous steroids, which lowers the content of cortisone-sensitive cells in the lymphoid organs. As demonstrated in Figure 5A, the applied peptides reversed the loss of cell content of the thymus in a comparable manner. 

Likewise, the peptides partially normalized the distorted peripheral blood cell picture characterized by a remarkable increase in neutrophil precursors and eosinophils in untreated mice with inflamed colons (Figure 5B). The peptides substantially reduced the content of neutrophil precursors and eosinophils, but not the total percentage of myeloid cells. As calculated by the ANOVA of the K-W test, these effects were significant in the case of the P03 peptide.

### 3.6. In Silico Activity and Target Prediction Analysis

#### 3.6.1. ADME-T Predictions and Beyond

The Lipinski rule of five helps in drug design and development to filter druggable molecules based on the number of H-bond donors (no more than five) and acceptors (no more than 10), molecular weight (<500 g/mol), molar refractivity (40–130), and logP (a higher value of LogP identifies lower absorption) [65,66,67]. The latter means lipophilicity, which describes the absorption rate in the human body. Notably, in silico predictions suggest that stereomers met the Lipinski rule of five criteria, with negligible deviation (molecular weight = 502.6), as well as Egan (−1.0 ≤ logP ≤ 5, and topological polar surface area, TPSA, ≤140 Å^2^), Muegge (pharmacophore filter), and Veber filters with zero violations. On the other hand, stereomers do not comply with the Ghose criteria, with three violations such as MW > 480, MR > 130, and ≠ atoms > 70. The bioavailability (drug) score is 0.55, which means preferable pharmacokinetic features. More specifically, drug score value takes into account drug-likeness, molecular weight, the logarithm of the partition coefficient (cLogP), and the logarithm of solubility (logS), as well as toxicity risks. As seen in Table S1, the calculations revealed that these parameters were satisfied: logP < 5 for all structures, Log S (solubility) < 4 (here, moderately soluble), and SA (synthetic accessibility) < 10 for all moieties. Moving forward, according to the ADME guidelines, the molecules indicate good intestinal absorption (TPSA < 140 Å^2^). TPSA is a predictor of molecule transport properties, such as intestinal absorption, bioavailability, and blood–brain barrier penetration. %ABS is a derived variable calculated on the base equation %ABS = 109 − (0.345 × TPSA) [68]. Values obtained close to 80% signify relatively good drug transport properties. Moreover, strereomers can be P-glycoprotein (P-gp) substrates and CYP3A4 inhibitors. It can be mentioned that P-gp acts as a bio-barrier by extruding toxins and xenobiotics out of cells. Compounds show high skin permeability. The Caco-2 cell monolayer is useful as an in vitro model of the intestinal mucosa for the prediction of absorption of orally administered drugs. In this regard, the majority of molecules present good values (>0.9). No compound has poor intestinal absorption (less than 30%), a predictor of the percentage of a drug absorbed by the intestine (the primary site for the absorption of a drug from an orally administered solution). Furthermore, the compounds do not behave as an OCT2 substrate. OCT2 is a renal uptake transporter that has relevance in drug disposition and renal clearance. Moreover, molecules do not exhibit an AMES toxic nature. In other words, given molecules do not have mutagenic potential. No compounds reveal skin sensitization. On the other hand, molecules can be hepatotoxic. All ADMET properties are summarized in Table S1, while the pkSCM values are included in Table S2. 

Bioavailability radars are a graphic way for compact representation of bioavailability data in terms of physicochemical properties such as lipophilicity, size, polarity, solubility, flexibility, and saturation. It can be appreciated that the analyzed compounds show satisfying results in terms of drug-likeness (Figure S8).

The BOILED-Egg model (brain or intestinal estimated) is helpful in either the evaluation or visualization of blood–brain barrier (BBB) penetrability and passive human gastrointestinal absorption of molecules [33]. The representative diagram for all analyzed steromers is generated based on WLOGP (lipophilicity) and TPSA (topological polar surface area) parameters (Figure S9). Thus, BOILED-Egg is suitable for estimating the drug-likeness of candidate molecules. Here, BOILED-Egg models were predicted to recognize the penetrability of analyzed molecules. As can be seen in Figure S9, stereomers occupy the white region in the model, which indicates that they can absorbed by the intestinal system. It can be mentioned that P-gp, known as a multidrug resistance protein, is key in drug transport to the organs in the body. Thus, it can be a determinant of the bioavailability and pharmacokinetic features of drugs (absorption, distribution, elimination, and drug–drug interactions) [33]. The blue and red points signify compounds predicted as actively effluxed from the central nervous system by P-gp (PGP+) and non-substrates of P-gp (PGP), respectively. To conclude, molecules do not tend to permeable through the brain.

**Figure 6:**
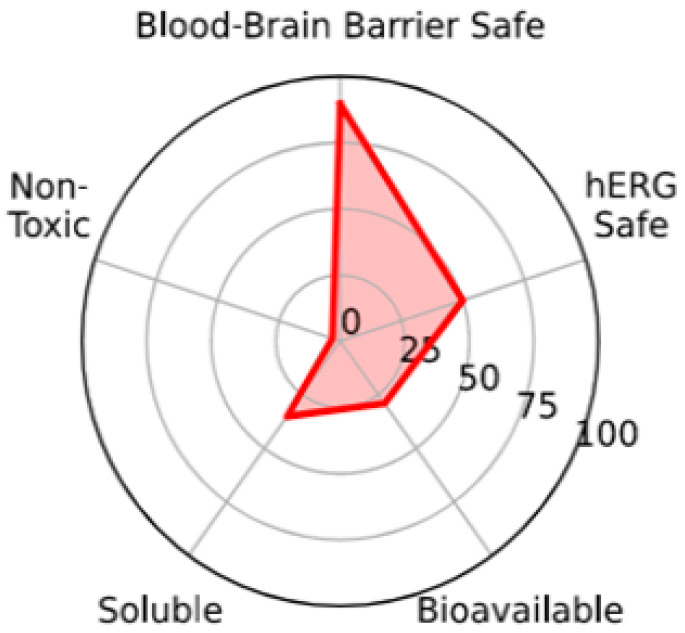
Radial plot representative for all analyzed stereomers.

Generally speaking, according to SwissADME predictions, stereomers show similar parameters with small differences in lipophilicity. Therefore, we also used the ADMET-AI web server to verify nuances. ADMET-AI is an open-source and easy-to-use machine learning platform that can serve as a powerful drug discovery tool for identifying compounds with favorable ADMET profiles for further development [35] (https://admet.ai.greenstonebio.com, accessed on 1 July 2024). Here, we observed greater differences in values (Table 4). Nevertheless, the general landscape of pharmacokinetic properties is similar, resulting in nearly the same radial plot (see Figure 6). This plot includes the following parameters: blood–brain barrier-safe (‘the probability that the molecule does not cross the blood–brain barrier’), hERG-safe (‘the probability that the molecule does not block the human ether-a-go-go channel’), bioavailable (‘the probability that the molecule is orally bioavailable’), soluble (‘the aqueous solubility of the molecule’), and non-toxic (‘the probability that the molecule is not toxic across all toxicity properties’).

#### 3.6.2. Toxicity Potential Assessment

According to Pro-Tox II, stereomers can be classified as relatively safe (toxicity class: 4; predicted LD50 = 1000 mg/kg). A toxicity risk assessment, performed by OSIRIS Data Warrior Software v. 6.1.0 [37], showed no mutagenic, tumorigenic, irritant, or reproductive toxicities. 

#### 3.6.3. Prediction of Peptide Activity

The activity of stereomers was predicted using the Peptide Ranker (http://distilldeep.ucd.ie/PeptideRanker, accessed 1 July 2024). This comprehensive peptide database for bioactivity potential scoring provides classes of biopeptides with specific structural features that accord their functions via different categories of peptides. There are common properties across diverse functional classes, which suggests that in silico prediction can accelerate across many classes. The molecules with a score of more than 0.5 can be considered bioactive. According to the rule, the higher a set threshold, the higher the anticipated activity [69]. Nevertheless, it should be highlighted that it is not a prediction of the degree of bioactivity. 

As a result of calculations, all stereomers are bioactive. It should be highlighted that P00, as well as P03—the compound that is the main subject of this article—and P04, has the highest score (greater than 0.97), while P06 has the lowest value (0.87).

#### 3.6.4. Bioactivity Score Prediction

The bioactivity score is a measure of the ability of compounds to interact with particular biological targets—the most common proteins. More specifically, it is a predictor of potential GPCR ligands, ion channel modulators, kinase inhibitors, nuclear receptor ligands, proteases, and enzyme inhibitors. The parameters were calculated using Molinspiration software. It is known that the molecule is active when the score is more than 0.0; moderately active when the score is between −5.0 and 0.0; and inactive when the value is less than −5.0. As seen in Table 5, analyzed molecules display the highest bioactivity scores as protease inhibitors (0.52) and GPCR ligands (0.33). Moreover, they show positive values as ion channel modulators and enzyme inhibitors. 

#### 3.6.5. PASS Analysis

PASS (Prediction of Activity Spectra for Substances) software (Institute of Biomedical Chemistry, Moscow, Russia) predicts more than 4000 types of bioactivities related to pharmacological effects, adverse effects, toxicity, biochemical mechanisms of action, interactions with metabolic enzymes and transporters, influences on gene expression, and so on. This prediction is correlated with the analysis of structure–activity relationships for over 250,000 bio-active molecules, including drugs and drug candidates but also toxic substances [70,71,72]. PASS analysis may efficiently find either new targets or new ligands. The activity is estimated as Pa (probably active) and Pi (probably inactive). Moieties with a Pa greater than Pi are considered for a particular pharmacological activity.

The predicted Pa and Pi values for all analyzed molecules are summarized in Table 6. We selected the types of activity that were predicted for analyzed molecules with the highest probability (Pa > 0.55). All molecules show the same wide range of potential bioactivities, such as nootropics, uterine relaxants, nicotinic receptor antagonists, CYP2C12 substrates, chitinase inhibitors, glycopeptide-like antibiotics, interleukin 2 agonists, antineoplastics, membrane integrity antagonists, Na^+^ transporting two-sector ATPase inhibitors, and Somatostatin 2 agonists.

#### 3.6.6. CLC-Pred: Cell-Line Cytotoxicity Prediction

The in silico predictions of the cytotoxic activity of analyzed compounds with Pa > 0.4 for various cancer cell lines are collected in Table 7. The findings highlighted the highest potential of compounds against pancreatic carcinoma and adult B acute lymphoblastic leukemia. In addition, other potential activities of the analyzed molecules are possible, such as hematopoietic and lymphoid, cervix, blood, stomach, skin, blood, and ovarium cancers.

#### 3.6.7. Target Protein Identification

**Figure 7:**
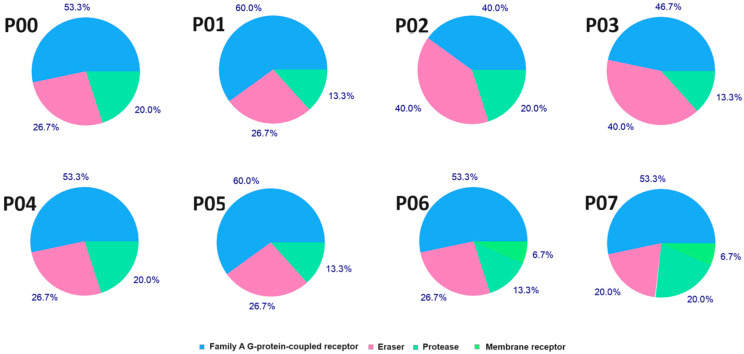
The diagrams represent the predicted biological molecular targets for the analyzed stereomers.

The free web server Swiss Target Prediction (http://www.swisstargetprediction.ch/, accessed on 1 July 2024) was used to identify potential molecular intracellular targets in terms of subjected stereomers [73]. Only the human (Homo sapiens) target protein was included in the data retrieval [74,75]. A graphical representation of likely biological targets is presented in Figure 7. Overall, the main interaction for all stereomers can mainly be GPCR (G protein-coupled receptor) ligands, proteases, and erasers, with few differences between them. In addition, membrane receptors are another highly probable class of targets for P06 and P07 molecules. More specifically, we predicted mainly Mu opioid receptor proteins (encoded as OPRM1), Delta opioid receptors (OPRD1), Oxytocin receptors (OXTRs) in the GPCR class of targets, Histone deacetylase (HDAC1/2), Histone deacetylase 3/nuclear receptor corepressor (NCOR2HDAC3) in the eraser class, and Thrombin (F2) in the protease class as potential targets for stereomers (Table S3).

### 3.7. NMR Studies

**Figure 8:**
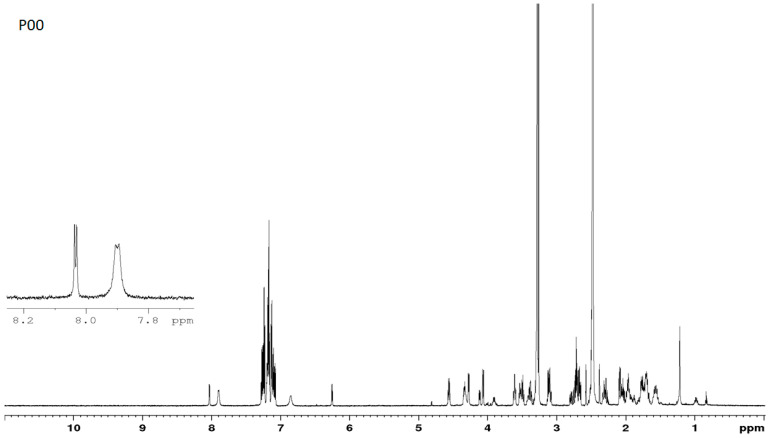
Representative ^1^H NMR spectrum for P00.

**Figure 9:**
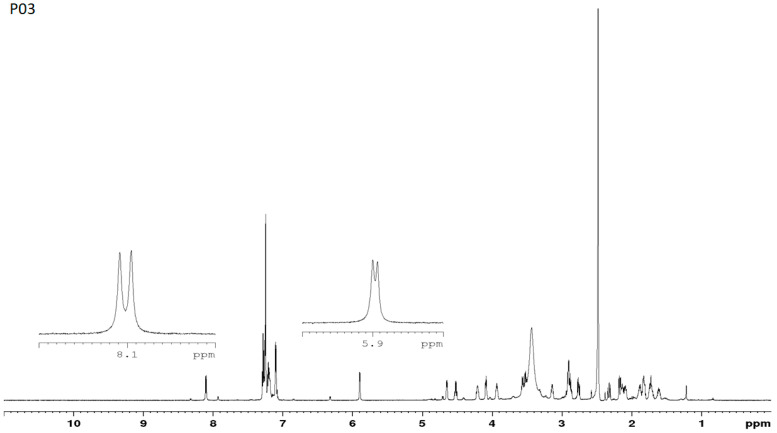
Representative ^1^H NMR spectrum for P03.

**Figure 10:**
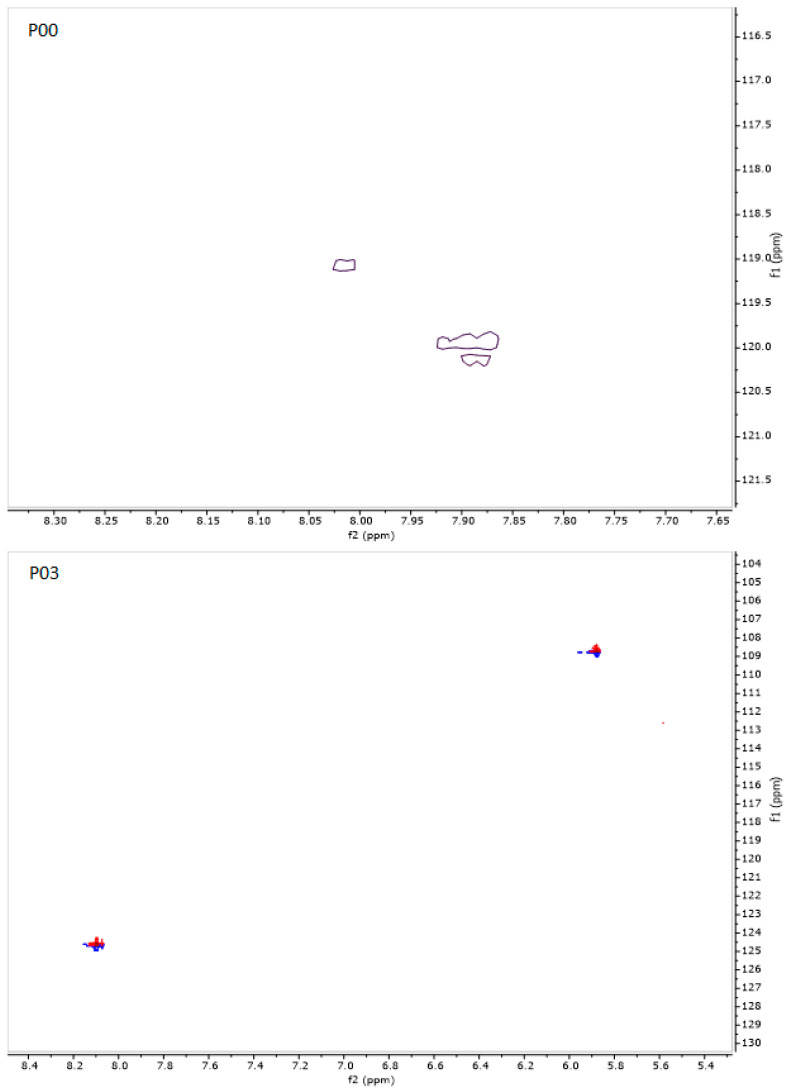
^1^H–^15^N HSQC 2D NMR spectra for P00 and P03.

Representative ^1^H NMR spectra for P00 and P03 are shown in Figure 8 and Figure 9. A brief comparison of the spectra allows us to see that they are significantly different, especially in the region of N-H protons. For the parent compound, where all chiral centers are L, two doublets were observed at δ = 7.89 ppm (Phe) and δ = 8.02 ppm (HoPhe) with vicinal ^3^J_H-H_ coupling constants equal to 7.91 and 4.81 Hz, respectively. In turn, D-amino acid replacements in the P03 sample caused chemical shifts of diagnostic N-H protons, which are significantly different. In this case, one doublet at δ = 8.10 ppm (Phe) and one doublet at δ = 5.88 ppm (HoPhe) were observed. The vicinal coupling constants equal 9.02 Hz and 4.86 Hz, respectively. A significantly lower chemical shift for the NH group in HoPhe may result from shielding this proton with an aromatic ring. Analysis conducted for ^15^N NMR spectra also indicates differences in the chemical shifts for both samples. In the case of P00, the chemical shifts for nitrogen are equal to δ = 119.07 ppm and δ =119.93 ppm, while for P03, they are δ = 108.70 ppm and δ = 124.59 ppm. The 2D ^1^H-^15^N NMR heteronuclear correlations are presented in Figure 10. The assignments of the main chemical shifts in the NMR spectra of P00 and P03 samples are shown in Table 8 and Table 9.

### 3.8. Results of Quantum-Chemical Calculations 

**Figure 11:**
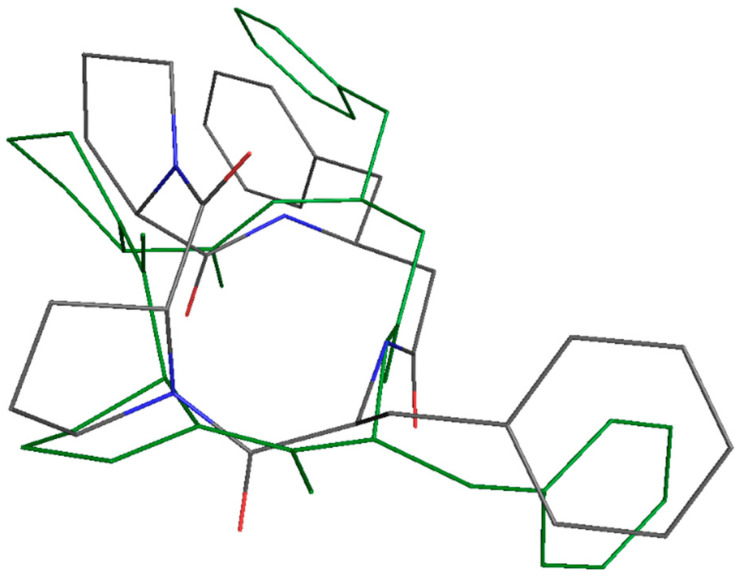
Superposition of the DFT-optimized structures of P00 (green) and P03 (C—gray, N—blue, O—red) in aqueous solutions.

The DFT-optimized structures of P00 and P03 in aqueous solutions (Figure 11, Tables S4 and S5) differ in torsion angles, which only causes insignificant changes in their electronic structures. Nevertheless, the Gibbs energy of P03 is significantly higher, its optical rotation is different, and it has a higher dipole moment (Table 10).

**Figure 12:**
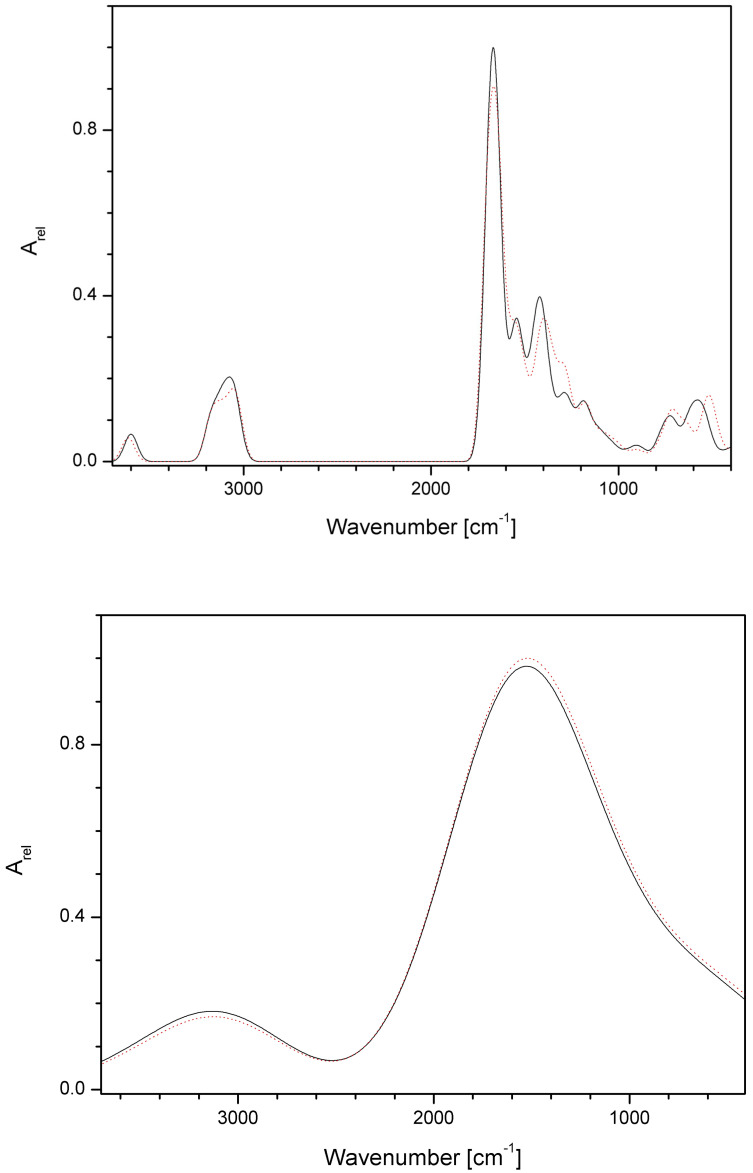
Calculated IR absorption spectra of P00 (full black line) and P03 (dotted red line) in aqueous solutions for FWHM = 0.01 eV (**top**) and 0.1 ev (**bottom**). The dimensionless relative absorbance A_rel_ is related to the highest P00 (**top**) or P03 (**bottom**) peaks.

**Figure 13:**
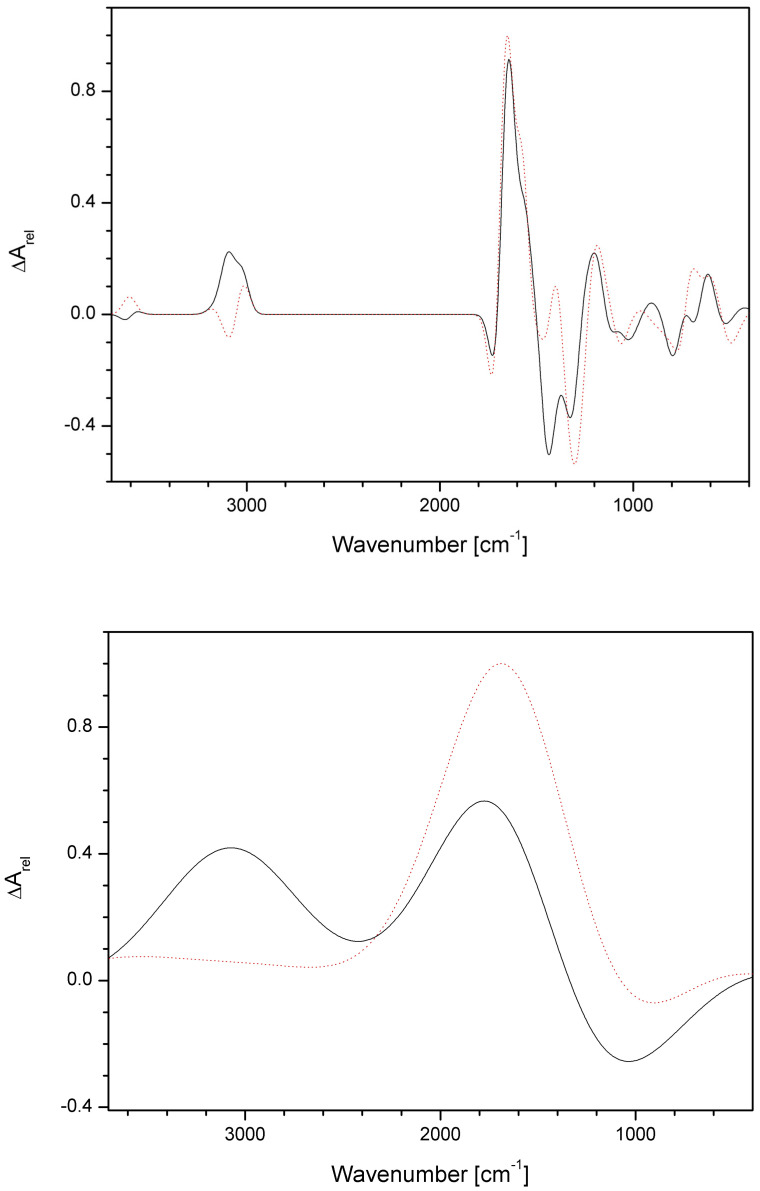
Calculated VCD spectra of P00 (full black line) and P03 (dotted red line) in aqueous solutions for FWHM = 0.01 eV (**top**) and 0.1 eV (**bottom**). The dimensionless relative absorbance A_rel_ is related to the highest P03 peaks.

IR and VCD spectra were calculated for all 207 vibration transitions of P00 and P03 in aqueous solutions using Gaussian widening of discrete lines with FWHM = 0.01 and 0.1 eV (Figure 12 and Figure 13). It is evident that despite the IR spectra being nearly identical for both compounds, the VCD spectra above ca 2500 cm^−1^ are able to distinguish between both compounds under study. For practical purposes, it would be necessary to find the optimal FWHM value (or use Lorentzian instead of Gaussian widening) between the values used in our study in order to obtain the highest possible similarity with experimental VCD spectra.

**Figure 14:**
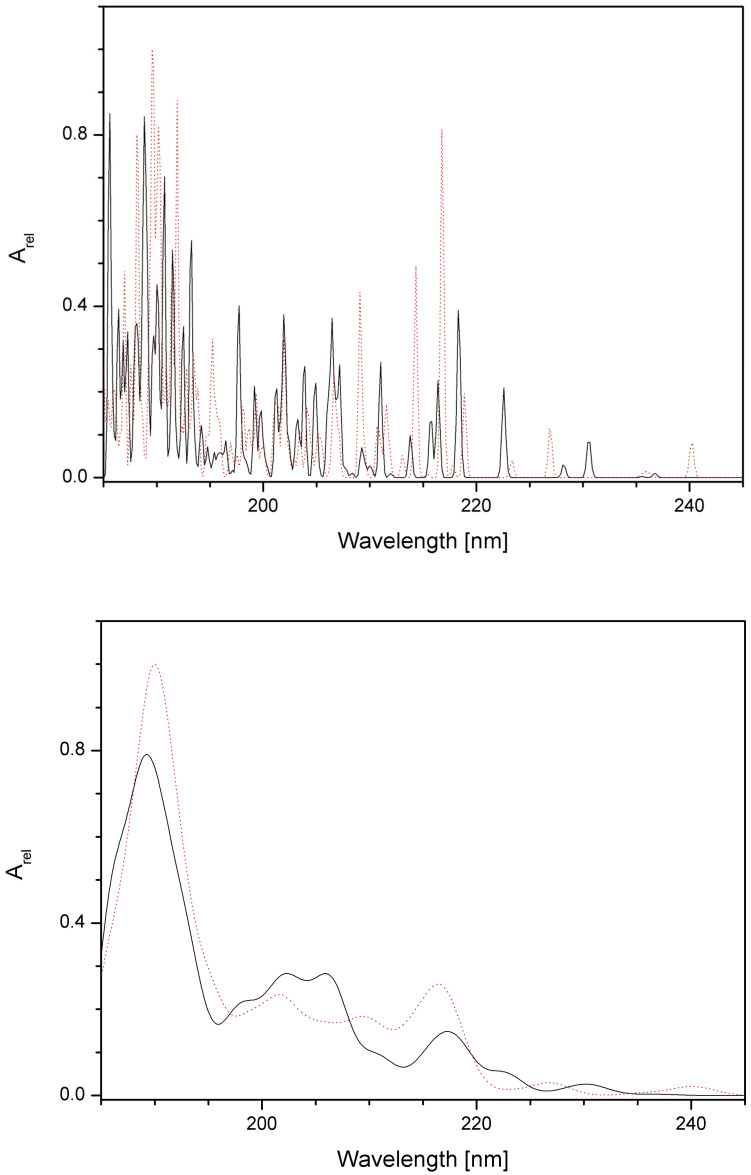
Calculated electron spectra of P00 (full black line) and P03 (dotted red line) in aqueous solutions for FWHM = 0.01 eV (**top**) and 0.1 eV (**bottom**). The dimensionless relative absorbance A_rel_ is related to the highest P03 peaks.

**Figure 15:**
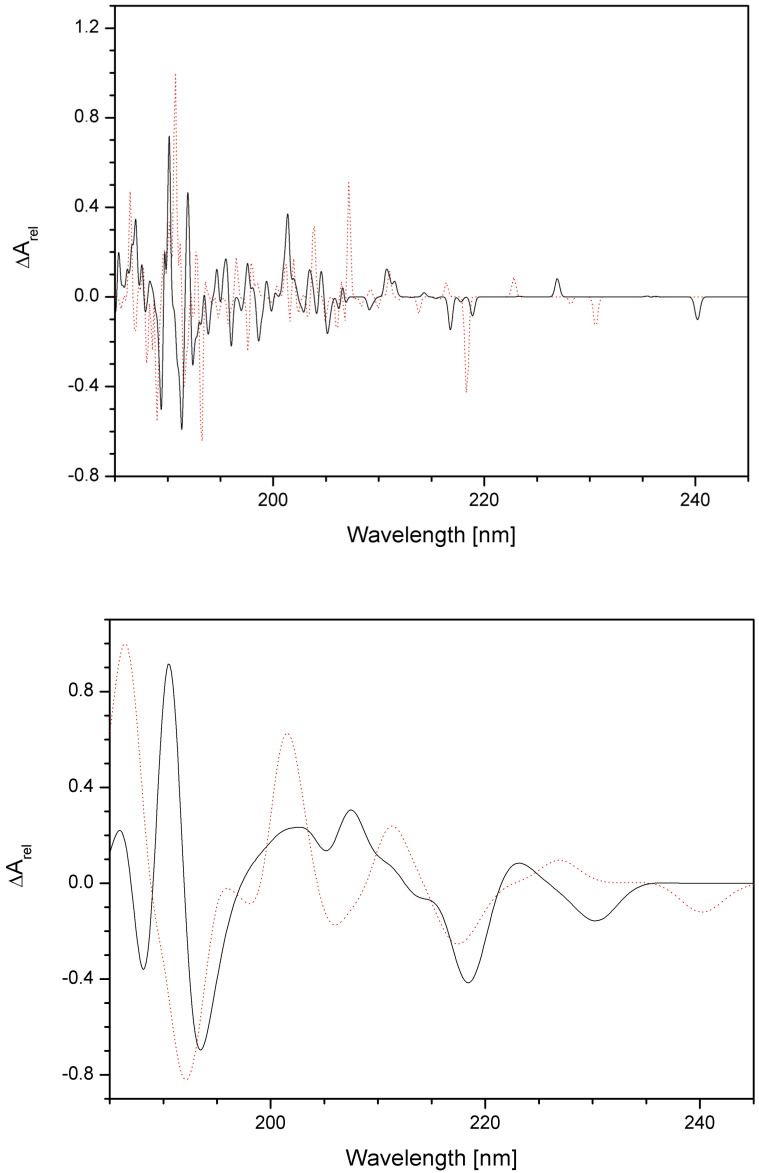
Calculated ECD spectra of P00 (full black line) and P03 (dotted red line) in aqueous solutions for FWHM = 0.01 eV (**top**) and 0.1 eV (**bottom**). The dimensionless relative absorbance A_rel_ is related to the highest P03 peaks.

The electronic and ECD spectra of P00 and P03 in aqueous solutions were calculated for the 100 lowest electron transitions (i.e., between 5.2 and 6.7 eV) with FWHM = 0.01 and 0.1 eV (Figure 14 and Figure 15). Although these spectra can give more information about both compounds, they are less suitable for their identification in solutions because of peak overlaps. In addition, in this case, it would be necessary to find the optimal FWHM value (or use Lorentzian instead of Gaussian widening) to obtain the highest possible similarity with experimental spectra.

The very near spectral lines of P00 and P03 reduce the information content due to high overlap, especially in ECD spectra, which makes them unusable for our purposes. A very recent study [76] has shown that the absolute configuration of cyclic peptides with four chiral centers cannot be unambiguously assigned using VCD. The authors concluded that other techniques are necessary for this purpose. They underlined that the CD spectra of all stereoisomers should be computed before an attempt to analyze experimental spectra; otherwise, accidental erroneous assignment is highly probable. On the basis of our (TD-)DFT calculations, we concluded that experimental ECD and VCD spectra cannot contribute to the assignment of the absolute configuration of our systems with four chiral centers. Therefore, their measurement is useless.

## 4. Discussion

This work shows that D-amino acid replacements in the 4B8M parent compound (Table 1), containing only L-amino acids, may lead to obtaining modified compounds with a more potent or different activity than the parent peptide. The studied cyclic tetrapeptides were nontoxic (Figure 1) and demonstrated a differential ability to suppress LPS-induced TNF-α production in the human whole blood cell model (Table 2). The peptides were virtually devoid of the anti-proliferative action, except for compound P03 at a concentration of 100 µg/mL (Figure 2). The lack of cell toxicity of the compounds at the highest dose (100 µg/mL) suggests that the anti-proliferative or suppressive actions in LPS-induced TNF-α production were not due to cytotoxic effects of the peptides on lymphocytes. 

The approach of replacing L-amino acids with D-amino acids in the parent peptide led to changes in the activity of the studied peptides (Table 3). It appears that some replacements may be advantageous to enhancing overall immunosuppressive activity and this relates to the presence of phenylalanine in the 4B8M peptide. However, in order to expose only one peptide property, such as suppression of TNF-α production, only replacement with D-proline appeared to be beneficial since the P04 peptide inhibited TNF-α levels more strongly than P03 (Table 3). We were of the opinion that the P03 peptide was of particular interest since it inhibited both cell proliferation and TNF-α production (Table 2, Figure 2), so the peptide was subjected to further investigations in in vivo mouse models, in parallel with the 4B8M peptide. Of note (unpublished), P03, at a concentration of 100 μg/mL inhibited the growth of some tumor cell lines such as cervix squamous cell carcinoma A431 (by 15%) and murine leukemia L1210 (by 34%). 

To reveal any therapeutic benefit of the new compounds, testing the compounds in in vivo experimental models is essential. In vivo models reduce some of the limitation factors seen in vitro, such as poor solubility and the undesirable effects of a solvent (vehicle). Our studies with 4B8M have revealed very potent anti-inflammatory actions in several in vivo mouse models [11]. It seems highly plausible, considering very similar immunosuppressive properties in the in vitro models, that the mechanism of action of P03 is similar to that of 4B8M [11], which regulates the production of cyclooxygenase-2 (COX-2) and expression of prostaglandin E2 (PGE2) receptors.

Therefore, we compared the anti-inflammatory properties of P03 with 4B8M in several in vivo mouse models relating to some clinical situations: specific contact sensitivity, rheumatoid arthritis, and nonspecific inflammation of the colon (Figure 3, Figure 4 and Figure 5). By the analysis of selected parameters in a given experimental model, supported with adequate statistical methods, we were able to demonstrate a predominance of P03 immunosuppressive activity over the 4B8M peptide. It is worth mentioning that the cyclic tetrapeptides used in our studies are therapeutically effective when administered in different routes, such as intraperitoneal, topical, and even oral, as shown for 4B8M and P03 in the currently presented studies and for 4B8M in the mouse model of ovalbumin (OVA)-induced lung inflammation [11]. The case of P03 is in agreement with another study proving that the cyclization and introduction of D-amino acids into a linear inhibitor of the forkhead box P3 (FOXP3) transcription factor may enhance ligand–receptor binding affinity [77].

In addition, we used diverse modern computational tools supported by artificial intelligence to check differences in the bio-pharmacokinetic parameters of the analyzed stereomers, which have gained significant interest in either industrial or academic settings in recent years. SwissADME or pkCSM web servers belong to the most popular in silico tools for the prediction of pharmacokinetic profiles. Nevertheless, they have limited capabilities in the calculation of larger structures. What is more, a differentiation of subtle differences in stereomers is also problematic. We tested new more advanced artificial intelligence-based tools, enabling more accurate and comprehensive analyses that are gaining more and more attention recently. This work indicates that careful and critical using of several (not one) freely accessible in silico tools is key. The current developments of machine learning supported by artificial intelligence undoubtedly show promise in predictions in biomedical chemistry, but further improvements are needed to ensure pharmacovigilance and the safety of drug candidates.

## 5. Conclusions

In summary, we successfully synthesized a new stereochemical variant of 4B8M, called P03. This study showed that selective replacements of single amino acids with D-isomers in a cyclic [Pro-Pro-β3-HoPhe-Phe-] tetrapeptide may lead to obtaining modified derivatives showing stronger or modified biological activities. 

Furthermore, based on in silico predictions, it is proposed that new stereomers, especially P03, have satisfactory ADME-T profiles, similar to 4B8M. Moreover, they may have therapeutic properties through newly predicted targets. The results show that stereomers might have diverse activities, including nootropic, antibiotic, or antineoplastic functions. New molecules are predicted as protease (and enzyme) inhibitors as well as GPCR receptors.

Quantum-chemical calculations indicate that the different activity of P00 and P03 is not due to their different electron structures. This difference can be ascribed rather to their geometry, which is reflected in the different optical activity of both compounds. According to our results, VCD spectra are most suitable for the identification of both compounds in aqueous solutions despite the fact that more information on pure compounds can be obtained from the ECD spectra. 

Finally, we hope that this work will provide helpful insight into the development of better therapeutic agents, keeping in mind immunosuppressive drugs. We also highlight the thoughtful use of computational tools. Machine learning methods supported by artificial intelligence are still in their infancy and need further development, especially in terms of larger structures and their stereochemical modifications.

## Figures and Tables

**Table 1:** Peptide labels and sequences form half of potential 4B8M stereomers (four chiral centers in a 13-element cycle give 16 possible stereomers).

Peptide	Sequence	
P00 (4B8M)	cyclo[Pro-Pro-*β*^3^-HoPhe-Phe-]	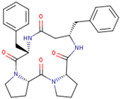
P01	cyclo[Pro-Pro-*β*^3^-HoPhe-*D*-Phe-]	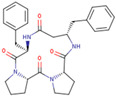
P02	cyclo- [*D*-Pro-*D*-Pro-*β*^3^-HoPhe-*D*-Phe-]	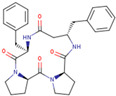
P03	cyclo-[*D*-Pro-Pro-*β*^3^-HoPhe-Phe-]	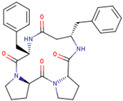
P04	cyclo-[Pro-Pro-*D*-*β*^3^-HoPhe-Phe-]	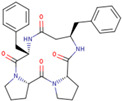
P05	cyclo-[Pro-*D*-Pro-*β*^3^-HoPhe-Phe-]	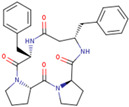
P06	cyclo-[*D*-Pro-*D*-Pro-*D*-*β*^3^-HoPhe-*D*-Phe-]	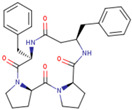
P07	cyclo-[*D*-Pro-*D*-Pro-*β*^3^-HoPhe-Phe-]	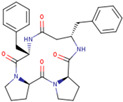

**Table 2:** Effects of P01–P07 peptides on LPS-induced TNF-α production in human whole blood cell cultures. Peptides P01–P07 and the reference compound 4B8M were tested at concentrations of 1 and 10 µg/mL. The cell culture without LPS, DMSO, and peptides is denoted as (-), and without DMSO and peptides as (LPS).

Compound Concentration (µg/mL)	TNF-α (pg/mL)	% of Inhibition vs. DMSO Control	Compound Concentration (µg/mL)
(-)	-	92	-
(LPS)	-	6668	-
DMSO	1	8167	-
	10	8006	-
P00 (4B8M)	1	7364	9.8
	10	7176	10.4
P01	1	7016	14.1
	10	5757	28.1
P02	1	6614	19.0
	10	6346	20.7
P03	1	6052	25.9
	10	5222	34.8
P04	1	5463	33.1
	10	5302	33.8
P05	1	7123	12.8
	10	7203	10.0
P06	1	8033	1.6
	10	7658	4.4
P07	1	6453	21.0
	10	7284	9.0

**Table 3:** Structure–activity relationship of the P01–P07 peptides.

Name	Structure	Suppressive Effect on Cell Proliferation	Suppressive Effect on TNF-α Production
P01	cyclo(Pro-Pro-*β*^3^-HoPhe-*D*-Phe-)	NS	marginal
P02	cyclo(*D*-Pro-*D*-Pro-*β*^3^-HoPhe-*D*-Phe-)	NS	small
P03	cyclo(*D*-Pro-Pro-*β*^3^-HoPhe-Phe-)	significant at 100 µg/mL	strong
P04	cyclo(Pro-Pro-*D*-*β*^3^-HoPhe-Phe-)	NS	strong
P05	cyclo(Pro-*D*-Pro-*β*^3^-HoPhe-Phe-)	NS	moderate
P06	cyclo(*D*-Pro-*D*-Pro-*D*-*β*^3^-HoPhe-*D*-Phe-)	NS	absent
P07	cyclo(*D*-Pro-*D*-Pro-*β*^3^-HoPhe-Phe-)	NS	small

**Table 4:** ADME-AI parameters for analyzed stereomers.

	P00	P01	P02	P03	P04	P05	P06	P07
**PHYSICOCHEMICAL** **Physicochemical**								
Molecular weight [Daltons]	502.62	502.62	502.62	502.62	502.62	502.62	502.62	502.62
Log P	1.83	1.83	1.83	1.83	1.83	1.83	1.83	1.83
H-bond acceptors	4	4	4	4	4	4	4	4
H-bond donors	2	2	2	2	2	2	2	2
Lipinski rule of 5	3	3	3	3	3	3	3	3
Quantitative estimate of drug-likeness	0.67	0.67	0.67	0.67	0.67	0.67	0.67	0.67
Stereo centers	4	4	4	4	4	4	4	4
Topological polar surface area (TPSA) [Å^2^]	98.82	98.82	98.82	98.82	98.82	98.82	98.82	98.82
**Absorption**								
Human intestinal absorption	0.58	0.62	0.62	0.52	0.54	0.59	0.61	0.56
Oral bioavailability	0.64	0.63	0.63	0.66	0.65	0.62	0.63	0.64
Aqueous solubility [log mol/L]	−3.80	−3.89	−3.91	−3.79	−3.75	−3.84	−3.87	−3.82
lipophilicity	1.88	1.97	1.97	1.85	1.83	1.90	1.93	1.88
Hydration free energy [kcal/mol]	−10.89	−10.82	−10.80	−10.91	−10.92	−10.85	−10.82	−10.87
Cell effective permeability [cm/s]	−5.41	−5.39	−5.39	−5.41	−5.41	−5.41	−5.40	−5.41
PAMPA permeability	0.50	0.52	0.52	0.50	0.51	0.51	0.52	0.50
P-glycoprotein inhibition	0.39	0.41	0.40	0.38	0.38	0.39	0.40	0.38
**Distribution**								
Blood–brain barrier penetration	0.16	0.16	0.16	0.16	0.15	0.15	0.16	0.16
Plasma protein binding rate [%]	82.16	83.23	83.34	81.84	81.55	82.57	82.77	82.28
Volume of distribution at steady state [L/kg]	−4.36	−4.25	−4.26	−4.40	−4.39	−4.32	−4.28	−4.35
**Metabolism**								
CYP1A2 inhibition	1.93 × 10^3^	2.31 × 10^3^	2.30 × 10^3^	1.69 × 10^3^	1.90 × 10^3^	2.17 × 10^3^	2.26 × 10^3^	1.92 × 10^3^
CYP2C19 inhibition	0.06	0.08	0.08	0.06	0.06	0.07	0.07	0.06
CYP2C9 substrate	0.14	0.14	0.14	0.14	0.13	0.14	0.14	0.14
CYP2C9 inhibition	0.03	0.04	0.03	0.02	0.02	0.03	0.03	0.03
CYP2D6 substrate	0.08	0.08	0.07	0.08	0.08	0.07	0.08	0.08
CYP2D6 inhibition	0.01	0.01	0.01	0.01	0.01	0.01	0.01	0.01
CYP3A4 substrate	0.77	0.78	0.78	0.77	0.78	0.77	0.79	0.77
CYP3A4 inhibition	0.37	0.43	0.42	0.33	0.36	0.40	0.42	0.36
**Excretion**								
Half-life [hr]	74.20	74.69	74.44	73.33	73.75	74.75	73.95	73.91
Drug clearance (hepatocyte) [μ L·min^−1^ (10^6^ cells)^−1^	29.25	30.80	31.02	29.01	28.38	29.64	30.18	29.53
Drug clearance (microsome) [mL·min^−1^·g^−1^]	51.72	54.14	54.42	51.25	50.71	52.47	53.48	52.09
**Toxicity**								
hERG blocking	0.29	0.31	0.31	0.28	0.29	0.30	0.32	0.29
Clinical toxicity	0.14	0.14	0.14	0.15	0.15	0.14	0.15	0.14
Mutagenicity	0.14	0.15	0.15	0.14	0.15	0.15	0.16	0.14
Drug-induced liver injury	0.65	0.67	0.67	0.64	0.64	0.66	0.66	0.65
Carcinogenicity	0.19	0.19	0.19	0.20	0.18	0.18	0.19	0.19
Acute toxicity LD50 [log(1/mol/kg)]	3.86	3.87	3.87	3.85	3.87	3.87	3.87	3.85
Skin reaction	0.210.12	0.21	0.21	0.20	0.21	0.21	0.22	0.21
Androgen receptor (full length)	0.03	0.12	0.12	0.11	0.12	0.12	0.12	0.12
Androgen receptor (ligand-binding domain)	0.01	0.03	0.04	0.03	0.04	0.04	0.04	0.03
Aryl hydrocarbon receptor	0.01	0.01	0.01	0.01	0.01	0.01	0.01	0.01
Aromatase	0.15	0.01	0.01	0.01	0.01	0.01	0.01	0.01
Estrogen receptor (full length)	0.03	0.15	0.15	0.15	0.15	0.16	0.16	0.15
Estrogen receptor (ligand-binding domain)	0.03	0.03	0.03	0.02	0.03	0.03	0.03	0.03
Peroxisome proliferator-activated receptor gamma	0.03	0.04	0.04	0.03	0.03	0.04	0.04	0.03
Nuclear factor (erythroid-derived 2)-like 2/antioxidant responsive element	0.25	0.28	0.28	0.23	0.26	0.27	0.28	0.25
ATPase family AAA domain-containing protein 5 (ATAD5)	0.02	0.03	0.03	0.02	0.02	0.03	0.03	0.02
Heat shock factor response element	0.03	0.04	0.04	0.03	0.03	0.04	0.04	0.03
Mitochondrial membrane potential	0.04	0.05	0.04	0.03	0.04	0.04	0.04	0.04
Tumor protein p53	0.09	0.11	0.11	0.08	0.09	0.11	0.11	0.09

**Table 5:** Bioactivity scores of analyzed compounds.

GPCR	Ion Channel Modulator	Kinase Inhibitor	Nuclear Receptor Ligand	Protease Inhibitor	Enzyme Inhibitor
0.33	0.02	−0.02	0.00	0.52	0.12

**Table 6:** Prediction of Activity Spectra for Substances—representative for all analyzed stereomers.

Pa	Pi	Activity
0.715	0.037	Nootropic
0.710	0.03	Uterine relaxant
0.702	0.026	Nicotinic alpha2beta2 receptor antagonist
0.702	0.057	CYP2C12 substrate
0.684	0.006	Chitinase inhibitor
0.678	0.004	Antibiotic glycopeptide-like
0.657	0.048	Nicotinic alpha6beta3beta4alpha5 receptor antagonist
0.610	0.005	Interleukin 2 agonist
0.585	0.017	Antineoplastic (non-Hodgin’s lymphoma)
0.577	0.028	Membrane integrity antagonist
0.555	0.056	Nicotinic alpha4beta4 receptor agonist
0.543	0.017	Na+ transporting two-sector ATPase inhibitor
0.509	0.003	Somatostatin 2 agonist

**Table 7:** CLC-pred values—representative for all stereomers.

Pa	Pi	Cell Line	Description	Tissue/Organ	Type	IAP *
0.582	0.005	PANC-1	Pancreatic carcinoma	Pancreas	Carcinoma	0.933
0.568	0.004	RS4-11	Adult B acute lymphoblastic leukemia	Bone marrow	Leukemia	0.835
0.496	0.015	RPMI-8226	Multiple myeloma	Hematopoietic and lymphoid tissue	Myeloma	0.883
0.486	0.044	HeLa	Cervical adenocarcinoma	Cervix	Adenocarcinoma	0.885
0.482	0.073	OCI-AML2	Adult acute myeloid leukemia	Blood	Leukemia	0.803
0.449	0.006	MKN-74	Gastric tubular adenocarcinoma	Stomach	Adenocarcinoma	0.856
0.426	0.032	SK-MEL-28	Melanoma	Skin	Melanoma	0.879
0.418	0.029	CCRF-CEM	Childhood T acute lymphoblastic leukemia	Blood	Leukemia	0.913
0.411	0.162	TMD8	Diffuse large B-cell lymphoma activated B-cell type	Lymphocytes	Lymphoma	0.811
0.406	0.027	CA46	Burkitts lymphoma	Blood	Lymphoma	0.810
0.403	0.212	A2780cisR	Cisplatin-resistant ovarian carcinoma	ovarium	carcinoma	0.838

*—IAP: Invariant Accuracy of Prediction (equal to AUC value) was calculated by a leave-one-out cross-validation procedure.

**Table 8:** The chemical shifts [ppm] for P00.

	Atoms															
Amino acids	C_α_	C_β_	C_β’_	C_γ_	C_δ_	Cε	Cζ	H_α_	H_β1_ H_β2_	H_β1’_ H_β2’_	H_γ1_ H_γ2_	H_δ1_ H_δ2_	Hε_1_ Hε_2_	Hζ_1_ Hζ_2_	NH	N
1Pro	63.36	30.97	-	21.56	47.39	-	-	4.12	1.97 1.77	-	1.74 1.58	3.60 3.29	-	-	-	-
2Pro	60.64	31.38	-	20.46	47.63	-	-	4.06	2.06 1.71	-	1.70 1.56	3.49 3.38	-	-	-	-
Phe	54.23	37.97	-	139.32	129.79	128.29	129.18	4.56	3.12 2.70	-	-	7.16	7.19	7.07	7.89	119.93
HoPhe	54.08	38.48	41.23	138.64	129.86	128.47	128.87	4.33	3.10 2.74	2.32 2.08	-	7.13	7.23	7.11	8.02	119.07

**Table 9:** The chemical shifts [ppm] for P03.

	Atoms															
Amino acids	C_α_	C_β_	C_β’_	C_γ_	C_δ_	Cε	Cζ	H_α_	H_β1_ H_β2_	H_β1’_ H_β2’_	H_γ1_ H_γ2_	H_δ1_ H_δ2_	Hε_1_ Hε_2_	Hζ_1_ Hζ_2_	NH	N
Pro	56.85	27.43	-	25.23	46.33	-	-	4.08	1.88 1.83	-	2.13 1.61	3.52 3.14	-	-	-	-
D-Pro	61.97	29.57	-	24.74	47.35	-	-	4.65	2.13 1.61	-	1.82 1.72	4.21 3.56	-	-	-	-
Phe	54.72	35.67	-	137.84	130.16	128.70	127.02	4.52	2.87 2.75	-	-	7.19	7.26	7.11	8.10	124.59
HoPhe	50.25	39.01	38.78	138.56	129.67	128.59	126.71	3.93	2.91 2.89	2.32 2.16	-	7.10	7.28	7.20	5.88	108.76

**Table 10:** Absolute (G_298_) and relative (ΔG_298_) Gibbs free energy at 298 K, specific static optical rotation [α]_D_, and dipole moments of the compounds under study.

Compound	P00	P03
G_298_ [Hartree]	−1645.48329	−1645.47100
ΔG_298_ [kJ/mol]	0.00	32.27
[α]_D_ [deg]	177.0	−30.3
Dipole moment [Debye]	7.04	12.09

## Data Availability

Data is contained within the article or Supplementary Material.

## Supplementary Materials

The following supporting information can be downloaded at: https://www.mdpi.com/article/10.3390/pharmaceutics16081106/s1, Figure S1. The L-stereoisomer (P00): (a) MS base peak chromatogram and MS spectrum; (b) UV chromatogram registered at 214 nm. The D-stereoisomer (P03): (c) MS base peak chromatogram and MS spectrum; (d) UV chromatogram registered at 214 nm; Figure S2. ^13^C spectrum of P03 sample; Figure S3. ^1^H-^1^H COSY spectrum of P03 sample; Figure S4. ^1^H-^13^C HSQC spectrum of P03 sample; Figure S5. ^13^C spectrum of P00 sample; Figure S6. ^1^H-^1^H COSY spectrum of P00 sample; Figure S7. ^1^H-^13^C HSQC spectrum of P00 sample; Figure S8. The bioavailability radar representative for all analysed stereomers; Figure S9. BOILED-Egg diagram for representative for all analysed stereomers; Table S1. The swissADME profile of analysed stereoisomers; Table S2. ADMET profile, representative for all analysed stereomers, according to calculation on pkCSM platform; Table S3. The predicted targets of stereoisomers by SwissTarget Prediction tool. (>0.2); Table S4. Coordinates (in Å) of the DFT optimized structure of P00 in aqueous solutions.
