# Supplementary material for: The Immunosuppressive Properties of Cyclo-[D-Pro-Pro-β3-HoPhe-Phe-] Tetrapeptide Selected from Stereochemical Variants of Cyclo-[Pro-Pro-β3-HoPhe-Phe-] Peptide
Source: Pharmaceutics. 2024 Aug 22;16(8):1106. doi: 10.3390/pharmaceutics16081106 (PMC11359963; doi:10.3390/pharmaceutics16081106)
Supplement: Supplementary file 1 [file pharmaceutics-16-01106-s001.zip › pharmaceutics-3138430-supplementary.pdf]

The Immunosuppressive Properties of Cyclo-[*D*-Pro-Pro-*β*³-HoPhe-Phe-] Tetrapeptide Selected from Stereo Chemical Variants of Cyclo-[Pro-Pro-*β*³-HoPhe-Phe-] Peptide

Krzysztof Kaczmarek*, Jolanta Artym, Joanna Bojarska*, Barbara Pacholczyk-Sienicka, Joanna Waśko, Ingrid Jelemenska, Wojciech M. Wolf, Martin Breza, and Michał Zimecki*

SUPPLEMENTARY INFORMATION

Materials and Methods

All solvents were HPLC or anhydrous p.a. grade (SIGMA-Aldrich, Milipore, ABI) and were used without further purification. Fmoc-D- and L-Pro-OH, Fmoc-D and L-*b*³-HomoPhenylalanine-OH, H-D- and L-Phe-2-Chlorotrityl resin, HBTU (2-(1 h-benzotriazol-1-yl)-1,1,3,3-tetramethyluronium hexafluorophosphate), HOBt (1-hydroxybenzotriazole), DIPEA were from Chem Impex, Inc. (Wooddale, IL) or IRIS Biotech (Germany). All other organic reagents were supplied by Aldrich.

General automated SPPS on ABI 433A Synthesizer:

(i) The H-D- or L-Phe 2-Chlorotrityl resin (0.25 mM) was initially swollen in NMP with vertexing for 10 min at rt (room temperature).

(ii) Fmoc removal: 20% piperidine in NMP 3x 2,5 min while vertexing, rt. Completeness of deprotection is checked by comparing the difference in the value of conductivity between the last deprotection with the preceding one with conductivity meter, and if this value is higher than was set up in programming, the machine repeats the deprotections (up to three more).

(iii) Washing 6x NMP.

(iv) Coupling: fourfold excess of Fmoc-AA-OH (1 mM, 4 equiv.) in the cartridge, to which HBTU/HOBt (3.6 equiv., 0.45 M in DMF) and DIPEA (7.2 equiv., 2.0 M in NMP) were added; solution in the cartridge was agitated with gentle stream on nitrogen for 10 min, followed by a transfer to the washed resin. The resulting suspension was vortexed 45 min at rt. The reaction vessel is then drained, and the resin is thoroughly washed with NMP three times while vertexing.

(v). Capping: a solution of 5ml NMP solution containing acetic acid anhydride/DIEA/HOBt (19 mL/9.5 mL/800mg diluted with NMP to 400 mL) was added to the resin and the reaction vessel was vortexed for 15 min, drained, at which point the resin was thoroughly washed with DCM six times.

The final deprotection after coupling the last Fmoc-AA was performed following step (i).

The cleavage of linear tetrapeptides from the resin was accomplished with a cocktail composed of 2,2,2-Trifluorethanol /Dichloromethane/Acetic Acid (14:6:1) and the filtrates containing the crude linear peptide were evaporated under reduced pressure. Evaporation of each peptide solution was repeated twice with the addition of a small amount of n-hexane. The semisolid crude products were dissolved in 0.1 N HCl and extracted with Ethyl Acetate to remove N-acetylated (capped) peptides. The identities of the analogs after cleavage from the resin were confirmed via MALDI-MS on a Kratos Kompact Probe MALDI-MS machine (Kratos Analytical, Ltd., Manchester, UK), which confirmed the presence of correct mass ions. The crude peptides were checked for their purity (HPLC).

Cyclisation

The purity of all crude linear tetrapeptide analogs was higher than 90%, so they were subjected to cyclisation. All cyclisations were performed in DCM in very high dilution (0.1 mM, in 1 liter DCM) with the aid of EDC (2 equivalents) in the presence of HOAt (1 equivalent and 2,4,6-Collidine 1 equivalent). All cyclisations were checked a few times in 2-4 hr intervals for completeness by HPLC trace from small, evaporated sample of reaction solution (1 mL). In the case of absence of the starting linear tetrapeptide DCM was evaporated off, the residue was dissolved in 100ml of Ethyl Acetate and washed consecutively with water (3×25 mL), .5 M HCl_{aq} (3x25mL) and 1M $KHCO_3$ (5x 25mL), then with brine (1x25mL). Ethyl Acetate extracts were dried and evaporated off. Crude cyclic tetrapeptides were purified on preparative Kromasil C8 column (10mm x 250mm) with a gradient 40-80% B in A (A 0.05% TFA/Water, B 80% Acetonitrile/Water acidified with 0.038% TFA). Fractions containing main peak were combined and partially evaporated to remove most of Acetonitrile, then freeze-dried.

FIGURES

a)

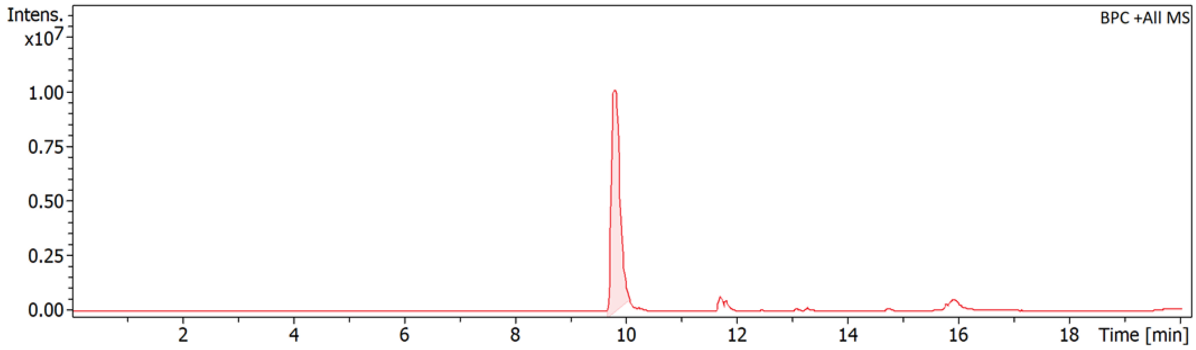

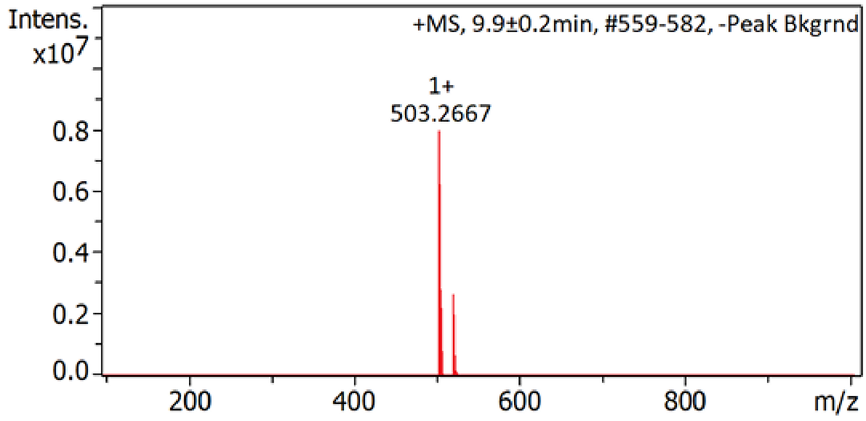
Intens.
x10^7
+MS, 9.9±0.2min, #559-582, -Peak Bkgrnd
1+
503.2667
0.8
0.6
0.4
0.2
0.0
200
400
600
800
m/z

b)

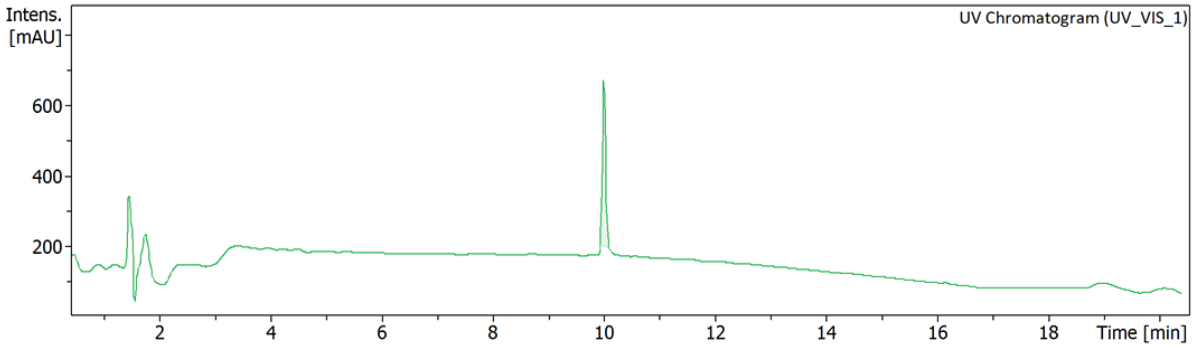
Intens.
[mAU]
UV Chromatogram (UV_VIS_1)
600
400
200
2
4
6
8
10
12
14
16
18
Time [min]

c)

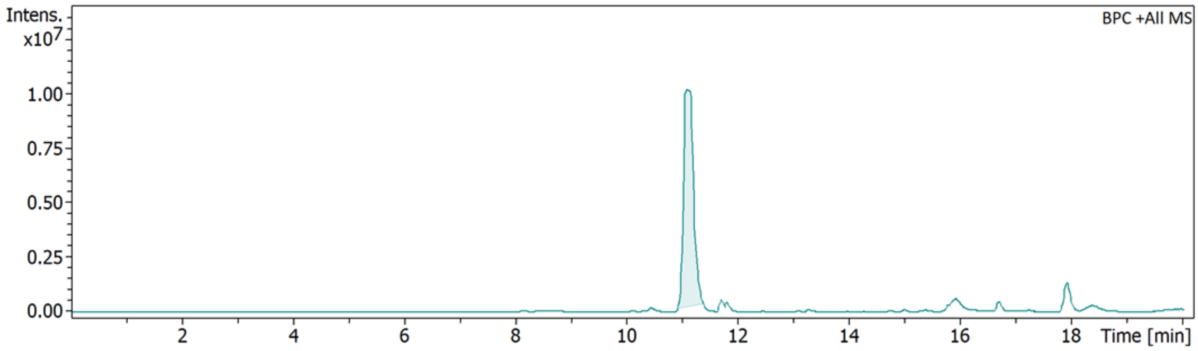
Intens.
x10^7
BPC +All MS
1.00
0.75
0.50
0.25
0.00
2
4
6
8
10
12
14
16
18
Time [min]

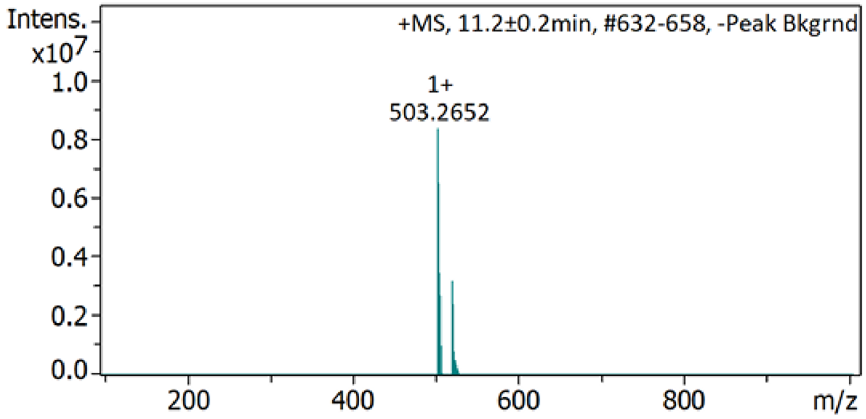
Intens.
x10^7
+MS, 11.2±0.2min, #632-658, -Peak Bkgrnd
1.0
1+
503.2652
0.8
0.6
0.4
0.2
0.0
200
400
600
800
m/z

d)

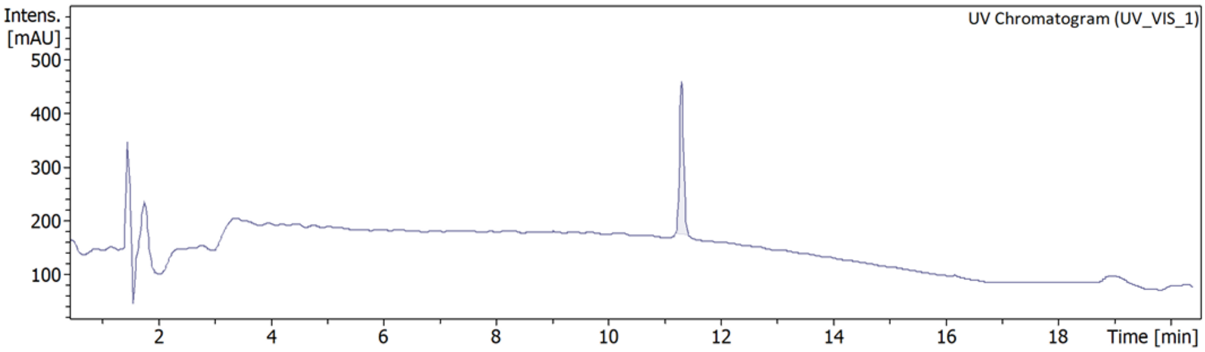

Figure S1. The L-stereoisomer (P00): a) MS base peak chromatogram and MS spectrum; b) UV chromatogram registered at 214 nm. The D-stereoisomer (P03): c) MS base peak chromatogram and MS spectrum; d) UV chromatogram registered at 214 nm.

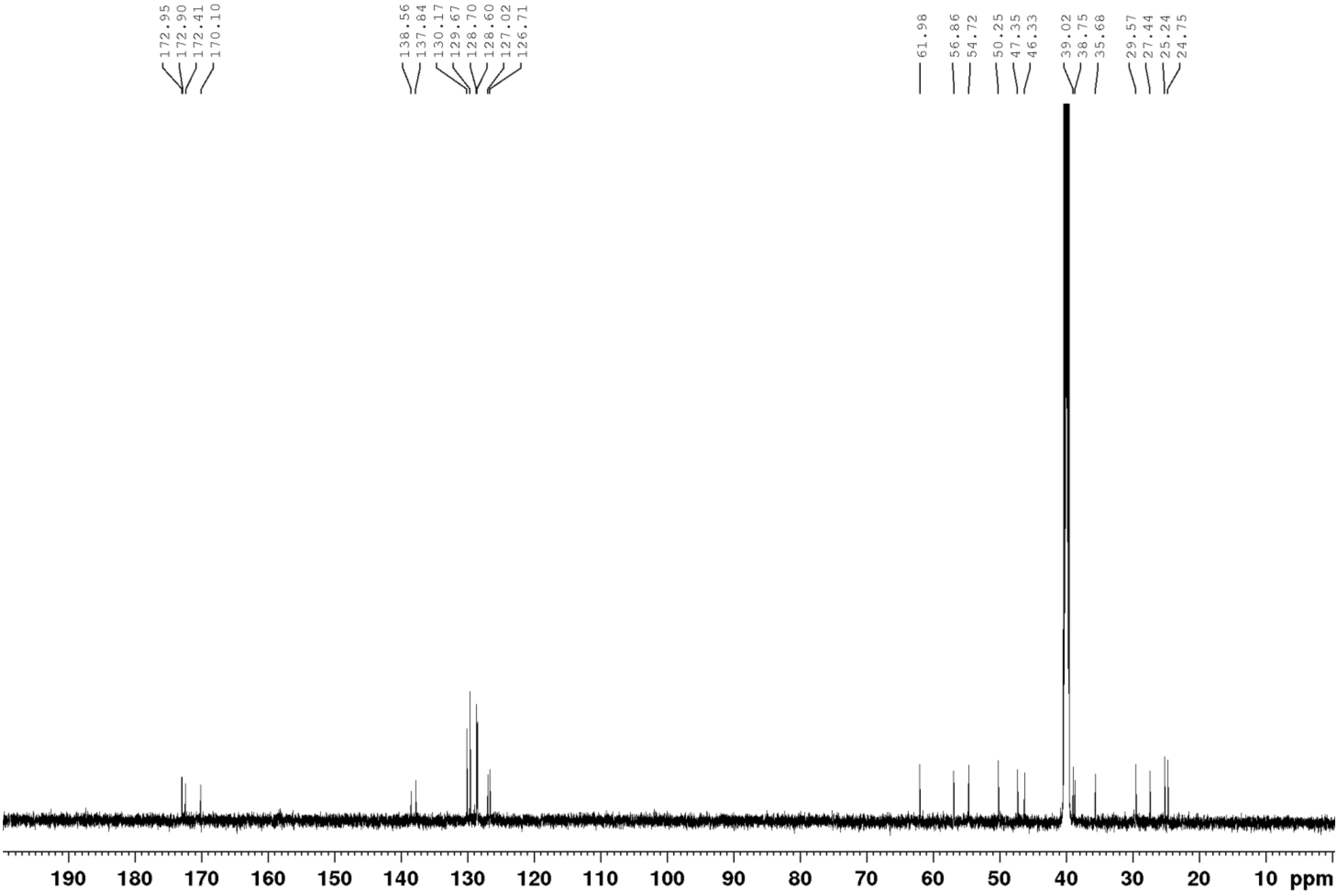

Figure S2. ^{13}C spectrum of P03 sample.

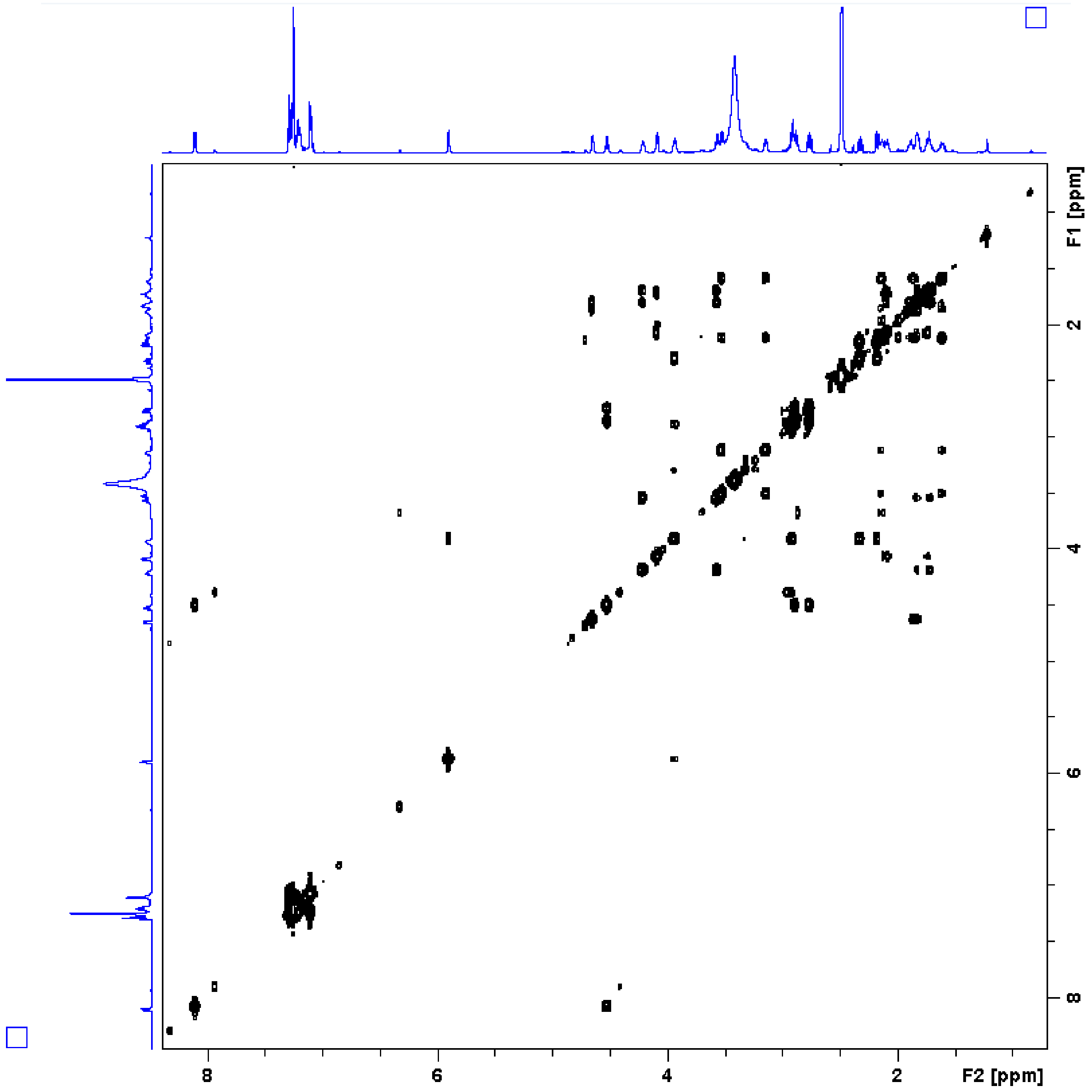

Figure S3. ^{1}H-^{1}H COSY spectrum of P03 sample.

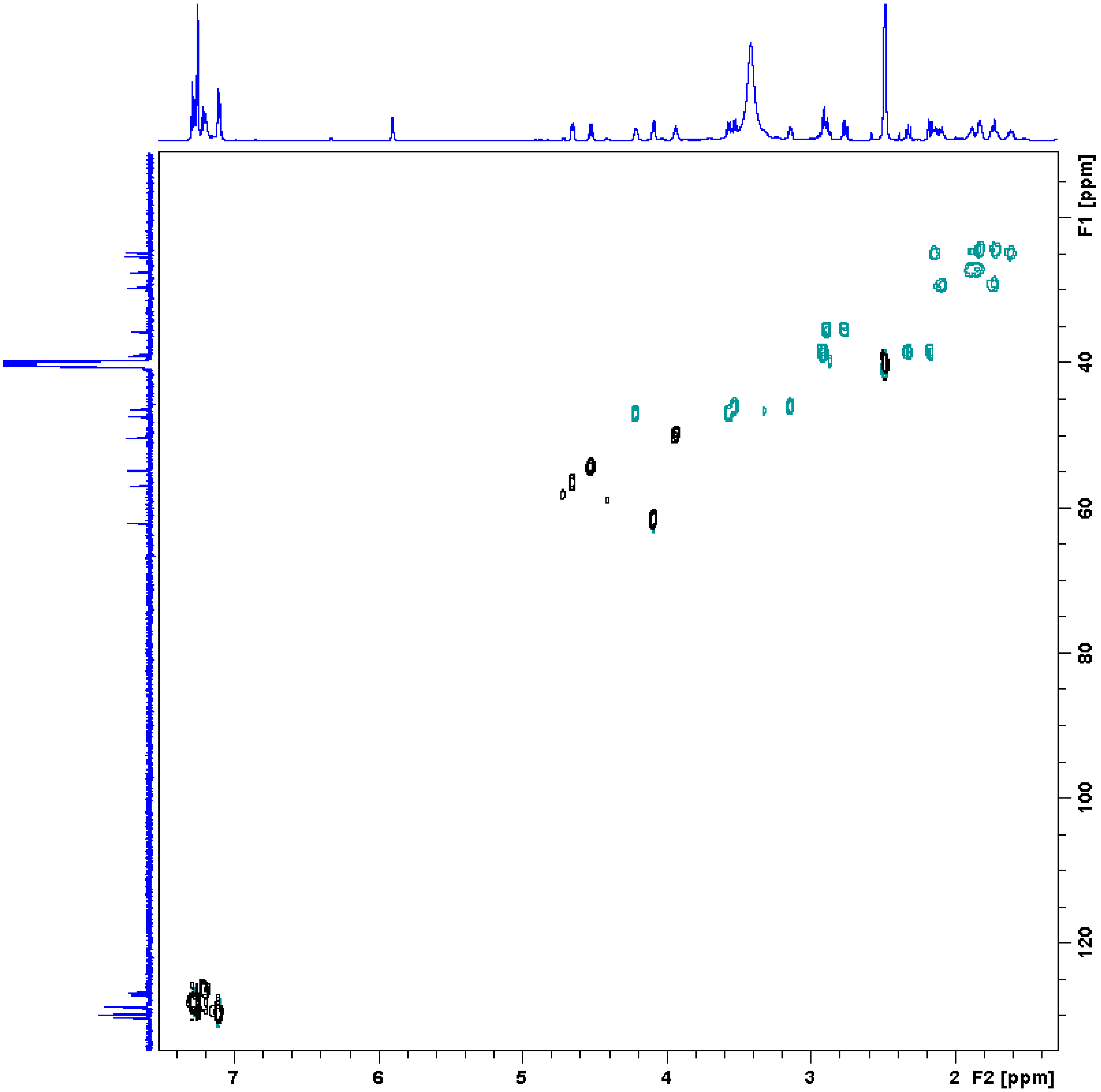

Figure S4. ^1H-^{13}C HSQC spectrum of P03 sample.

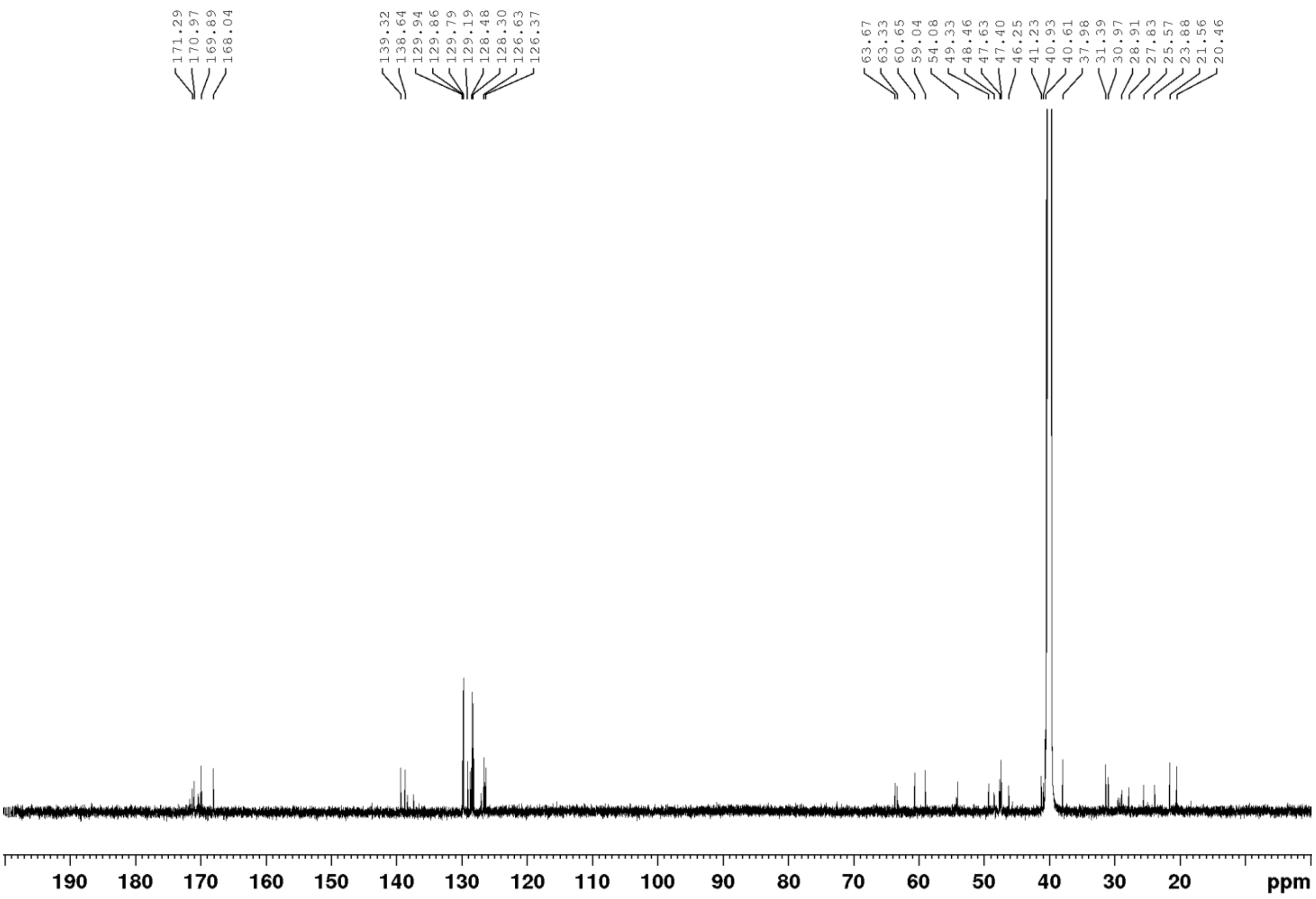

Figure S5. ^{13}C spectrum of P00 sample.

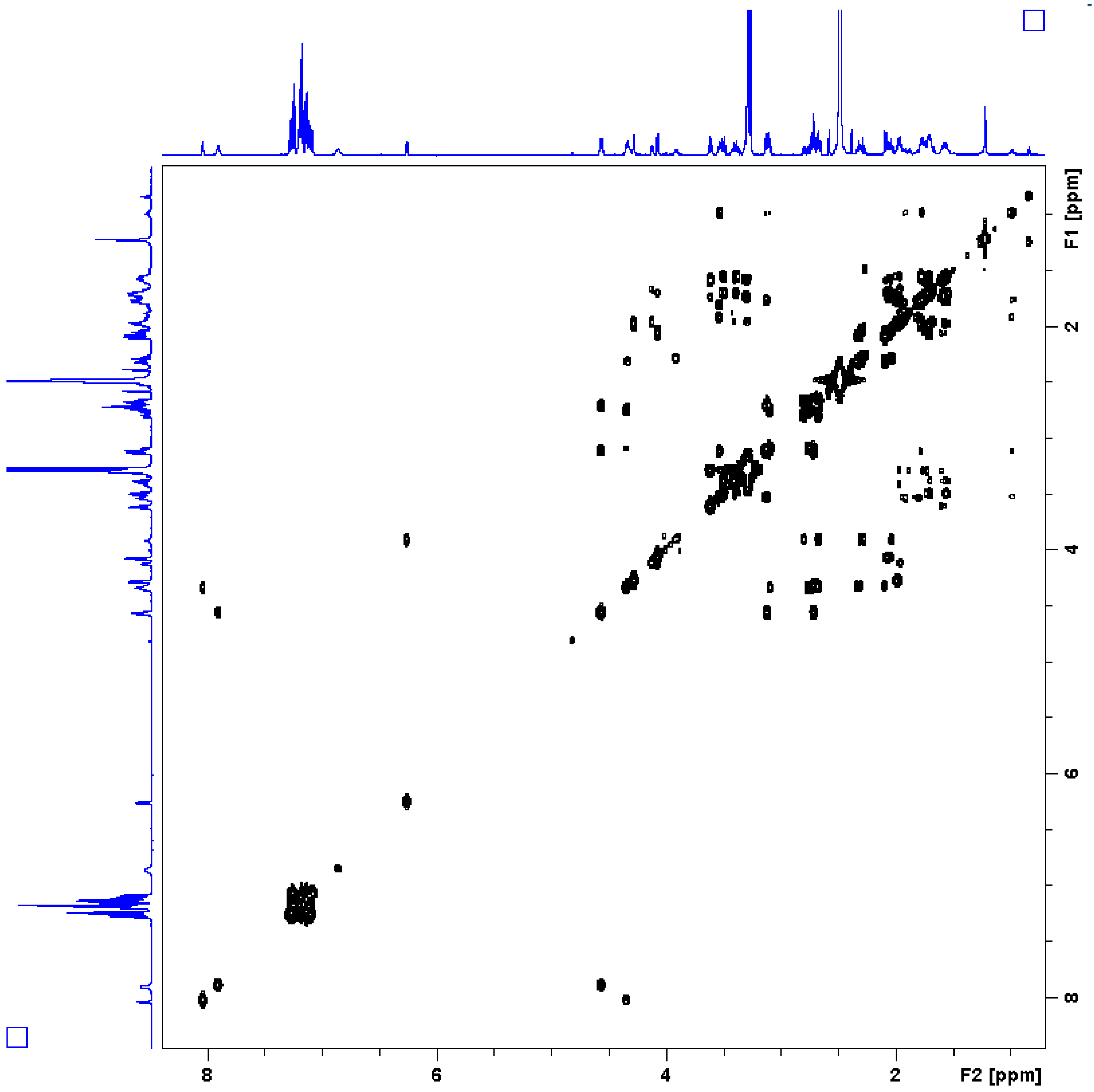

Figure S6. ^{1}H-^{1}H COSY spectrum of P00 sample.

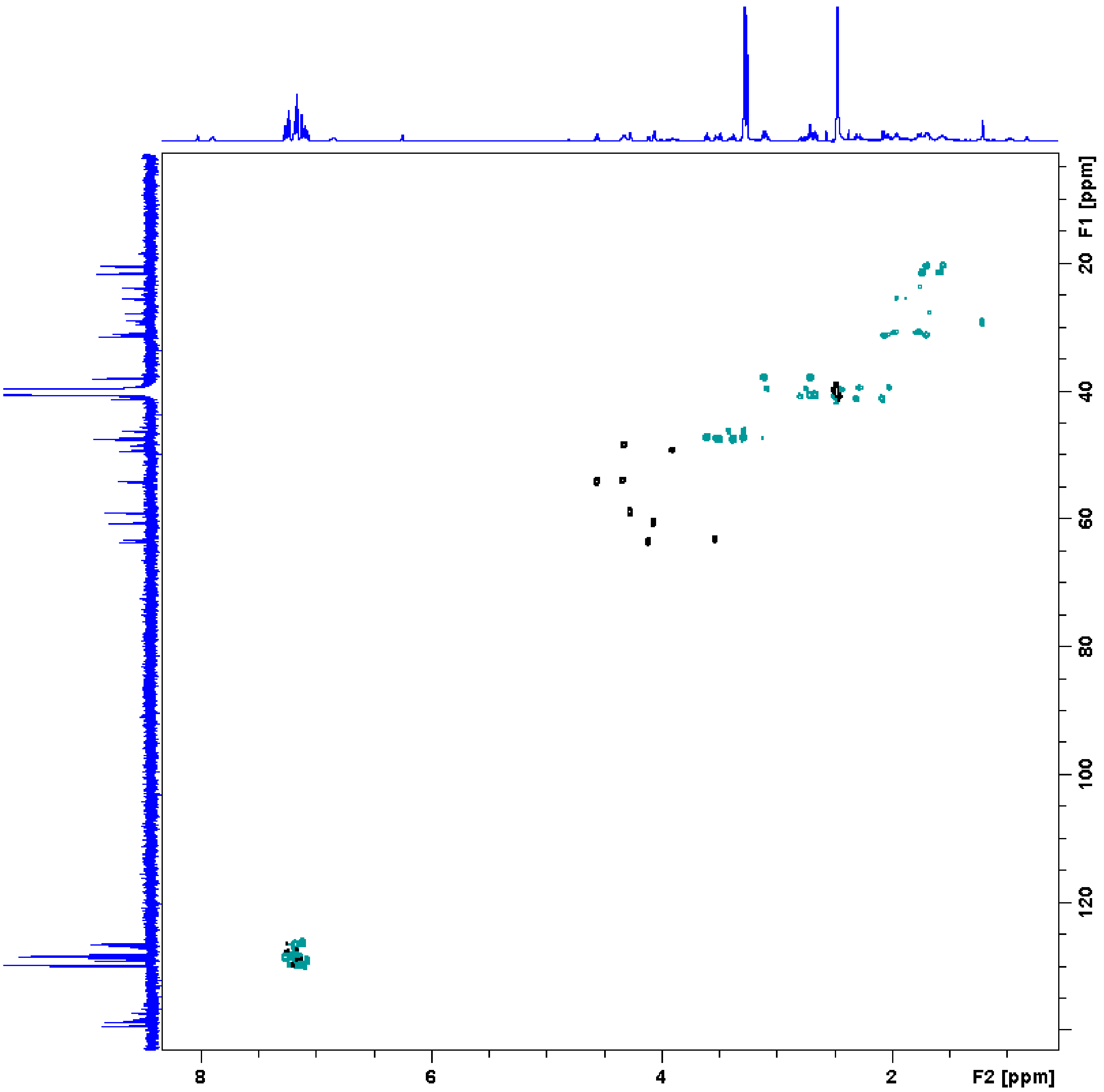

Figure S7. ^{1}H-^{13}C HSQC spectrum of P00 sample.

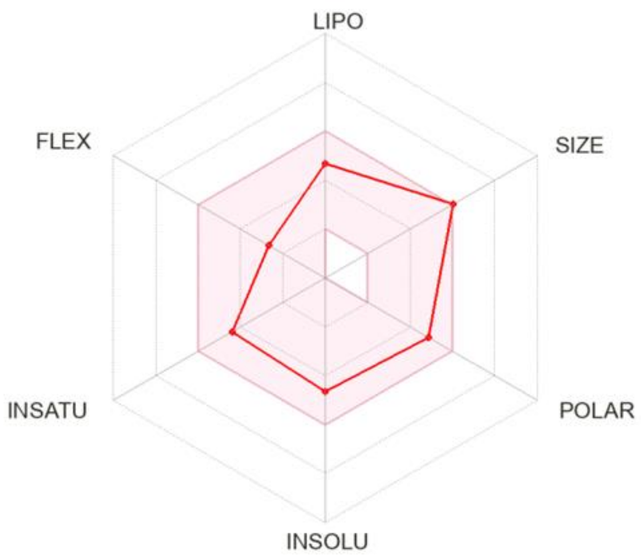

Figure S8. The bioavailability radar representative for all analysed stereomers.

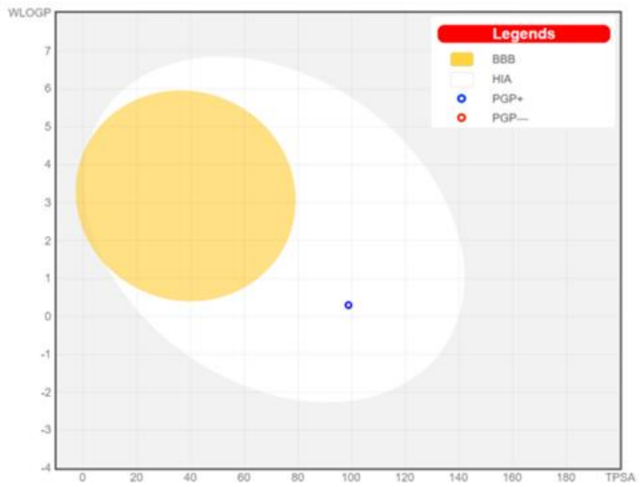

Figure S9. BOILED-Egg diagram for representative for all analysed stereomers

TABLES

Table S1. The swissADME profile of analysed stereoisomers.

Physicochemical properties	P00	P01	P02	P03	P04	P05	P06	P07
Formula	$C_{29}H_{34}N_4O_4$	$C_{29}H_{34}N_4O_4$	$C_{29}H_{34}N_4O_4$	$C_{29}H_{34}N_4O_4$	$C_{29}H_{34}N_4O_4$	$C_{29}H_{34}N_4O_4$	$C_{29}H_{34}N_4O_4$	$C_{29}H_{34}N_4O_4$
Molecular weight	502.60 g /mol	502.60 g /mol	502.60 g /mol	502.60 g /mol	502.60 g /mol	502.60 g /mol	502.60 g /mol	502.60 g /mol
No. heavy atoms	37	37	37	37	37	37	37	37
No. arom. heavy	12	12	12	12	12	12	12	12

atoms								
Fraction Csp³	0.45	0.45	0.45	0.45	0.45	0.45	0.45	0.45
No. rotatable bonds	4	4	4	4	4	4	4	4
No. H-bond acceptors	4	4	4	4	4	4	4	4
No. H-bond donors	2	2	2	2	2	2	2	2
Molar refractivity	154.32	154.32	154.32	154.32	154.32	154.32	154.32	154.32
TPSA [Å²]	98.82	98.82	98.82	98.82	98.82	98.82	98.82	98.82
%ABS = 109 – (0.345 x TPSA)	74.91	74.91	74.91	74.91	74.91	74.91	74.91	74.91
**Lipophilicity**								
LogPo/w (iLOGP)	3.01	3.18	2.69	3.80	3.33	3.19	3.35	2.28
LogPo/w (XLOGP3)	2.71	2.71	2.71	2.71	2.71	2.71	2.71	2.71
LogPo/w (WLOGP)	0.30	0.30	0.30	0.30	0.30	0.30	0.30	0.30
LogPo/w (MLOGP)	1.70	1.70	1.70	1.70	1.70	1.70	1.70	1.70
LogPo/w (SILICOS-IT)	2.32	2.32	2.32	2.32	2.32	2.32	2.32	2.32
Consensus LogPo/w	2.01	2.04	1.94	2.17	2.07	2.04	2.08	1.86
**Water solubility**								
LogS (ESOL)	-4.64	-4.64	-4.64	-4.64	-4.64	-4.64	-4.64	-4.64
Solubility [mg/ml; mol/l]	1.15e-02;2.29e-05	1.15e-02;2.29e-05	1.15e-02;2.29e-05	1.15e-02;2.29e-05	1.15e-02;2.29e-05	1.15e-02;2.29e-05	1.15e-02;2.29e-05	1.15e-02;2.29e-05
Class	Moder. Sol.	Moder. Sol.	Moder. Sol.	Moder. Sol.	Moder. Sol.	Moder. Sol.	Moder. Sol.	Moder. Sol.
LogS (Ali)	-4.44	-4.44	-4.44	-4.44	-4.44	-4.44	-4.44	-4.44
Solubility [mg/ml; mol/l]	1.83e-02;3.64e-05	1.83e-02;3.64e-05	1.83e-02;3.64e-05	1.83e-02;3.64e-05	1.83e-02;3.64e-05	1.83e-02;3.64e-05	1.83e-02;3.64e-05	1.83e-02;3.64e-05
Class	Moder. Sol.	Moder. Sol.	Moder. Sol.	Moder. Sol.	Moder. Sol.	Moder. Sol.	Moder. Sol.	Moder. Sol.
Log S (SILICOS-IT)	-7.12	-7.12	-7.12	-7.12	-7.12	-7.12	-7.12	-7.12
Solubility [mg/ml; mol/l]	3.85e-05;7.66e-08	3.85e-05;7.66e-08	3.85e-05;7.66e-08	3.85e-05;7.66e-08	3.85e-05;7.66e-08	3.85e-05;7.66e-08	3.85e-05;7.66e-08	3.85e-05;7.66e-08
Class	Poorly sol.	Poorly sol.	Poorly sol.	Poorly sol.	Poorly sol.	Poorly sol.	Poorly sol.	Poorly sol.
**Pharmacokinetics**								
GI absorption	High	High	High	High	High	High	High	High
BBB permeant	No	No	No	No	No	No	No	No
P-gp substrate	Yes	Yes	Yes	Yes	Yes	Yes	Yes	Yes
CYP1A2 inhibitor	No	No	No	No	No	No	No	No
CYP2C19 inhibitor	No	No	No	No	No	No	No	No
CYP2C9 inhibitor	No	No	No	No	No	No	No	No
CYP2D6 inhibitor	No	No	No	No	No	No	No	No
CYP3A4 inhibitor	Yes	Yes	Yes	Yes	Yes	Yes	Yes	Yes
LogKp (skin permeation) [cm/s]	-7.44	-7.44	-7.44	-7.44	-7.44	-7.44	-7.44	-7.44
**Druglikeness**								
Lipinski	Yes, 1 viol.:	Yes, 1 viol.:	Yes, 1 viol.:	Yes, 1 viol.:	Yes, 1 viol.:	Yes, 1 viol.:	Yes, 1 viol.:	Yes, 1 viol.:

	MW>500	MW>500	MW>500	MW>500	MW>500	MW>500	MW>500	MW>500
Ghose	No, 3 viol: MW>480, MR>130,≠ atoms>70	No, 3 viol: MW>480, MR>130,≠ atoms>70	No, 3 viol: MW>480, MR>130,≠ atoms>70	No, 3 viol: MW>480, MR>130,≠ atoms>70	No, 3 viol: MW>480, MR>130,≠ atoms>70	No, 3 viol: MW>480, MR>130,≠ atoms>70	No, 3 viol: MW>480, MR>130,≠ atoms>70	No, 3 viol: MW>480, MR>130,≠ atoms>70
Veber	yes	yes	yes	yes	yes	yes	yes	yes
Egan	yes	yes	yes	yes	yes	yes	yes	yes
Muegge	yes	yes	yes	yes	yes	yes	yes	yes
Bioavailability score	0.55	0.55	0.55	0.55	0.55	0.55	0.55	0.55
**Medicinal Chemistry**								
PAINS	0 alert	0 alert	0 alert	0 alert	0 alert	0 alert	0 alert	0 alert
Brenk	0 alert	0 alert	0 alert	0 alert	0 alert	0 alert	0 alert	0 alert
Leadlikeness	No, 1 viol: MW>350	No, 1 viol: MW>350	No, 1 viol: MW>350	No, 1 viol: MW>350	No, 1 viol: MW>350	No, 1 viol: MW>350	No, 1 viol: MW>350	No, 1 viol: MW>350
Synthetic accessibility	4.85	4.85	4.85	4.85	4.85	4.85	4.85	4.85

Table S2. ADMET profile, representative for all analysed stereomers, according to calculation on pkCSM platform.

ABSORPTION	
Water solubility (log mol/L)	-4.161
Caco2 permeability (log Papp in 10^{-6} cm/s)	0.966
Intestinal absorption (human) (%)	74.579
Skin permeability (log Kp)	-2.902
P-glycoprotein substrate	yes
P-glycoprotein I inhibitor	yes
P-glycoprotein II inhibitor	yes
DISTRIBUTION	
VDss (human) (log L/kg)	0.227
Fraction unbound (human) (Fu)	0.169
BBB permeability (log BB)	-0.544
CNS permeability (log PS)	-2.555
METABOLISM	
CYP2D6 substrate	No
CYP3A4 substrate	Yes
CYP1A2 inhibitor	No
CYP2C19 inhibitor	No
CYP2C9 inhibitor	No
CYP2D6 inhibitor	No
CYP3A4 inhibitor	yes

EXCRETION	
Total clearance (log ml/min/kg)	0.721
Renal OCT2 substrate	no
TOXICITY	
AMES test	no
Max. Tolerated dose (human) (log mg/kg/day)	-0.914
hERG I inhibitor	no
hERG I inhibitor	yes
Oral rat acute toxicity (LD50) (mol/kg)	2.373
Oral rat chronic toxicity (LOAEL) (log mg/kg bw/day)	2.224
hepatotoxicity	yes
Skin sensitisation	no
T. Pyriformis toxicity (log ug/L)	0.314
Minnow toxicity (log mM)	2.969

Table S3. The predicted targets of stereoisomers by SwissTarget Prediction tool. (> 0.2)

**Gene**	**Target (target class)**	**Prediction probability**							
		P00	P01	P02	P03	P04	P05	P06	P07
OPRM1	Mu opioid receptor (G-PCR)	0.40	0.36	0.43	0.41	0.37	0.46	0.41	0.37
OPRD1	Delta opioid receptor (G-PCR)	0.40	0.36	0.31	0.35	0.31	0.33	0.41	0.35
HDAC1	Histone deacetylase 1 (eraser)	0.26	0.24	0.30	0.31	0.25	0.26	0.29	0.25
HDAC2	Histone deacetylase 2 (eraser)	0.19	0.19	0.19	0.20		0.24	0.18	0.21
NCOR2 HDAC3	Histone deacetylase 3 / nuclear receptor corepressor 2 (eraser)		0.19	0.18	0.20		0.24	0.18	0.21
F2	Thrombin (protease)	0.23	0.18	0.24	0.26	0.24	0.22	0.23	0.23
OXTR	Oxytocin receptor (G-	0.21	0.25	0.22	0.26	0.25	0.28	0.24	0.27

	PCR)								
AVPR1A	Vasopressin V1a receptor (G-PCR)	0.17	0.25	0.20		0.20	0.28	0.22	0.23
AVPR2	Vasopressin V2 receptor (G-PCR)		0.18				0.20		
OPRK1	Kappa opioid receptor (G-PCR)	0.19	0.18	0.21	0.20		0.22	0.20	0.19
CAPN1	Calpain 1 (protease)	0.19	0.19	0.21	0.20	0.20	0.19	0.20	0.21
TACR1	Neurokinin 1 receptor (G-PCR)	0.18	0.17	0.19			0.20	0.22	0.22
GALR2	galanin receptor 2 (G-PCR)	0.19	0.17			0.20	0.19	0.19	0.18
GALR1	galanin receptor 1 (G-PCR)	0.19	0.17			0.20	0.19	0.19	0.18

Table S4. Coordinates (in Å) of the DFT optimized structure of P00 in aqueous solutions.

8	4.286141	2.172830	-0.936796
1	1.796138	-0.224421	-1.811906
8	0.230665	3.159053	-1.668384
1	-1.563861	0.325870	-1.032778
7	2.300645	2.664624	-0.015768
7	-1.420000	2.482932	-0.280745
8	1.128743	-1.424917	1.028378
7	1.864577	-0.302185	-0.806584
8	-2.055183	-0.272207	2.024922
7	-1.680180	-0.279025	-0.230983
6	-0.106038	2.698905	-0.580007
6	2.666131	4.100147	0.006727
1	2.695060	4.507630	-1.004866
1	3.665339	4.190753	0.435900
6	3.956002	-2.962433	-1.034047
1	3.647789	-2.694002	-2.039290
6	0.878577	3.593824	1.581498
1	-0.138615	3.840576	1.882262
1	1.430520	3.277841	2.469581
6	4.762311	-2.334860	1.142513
1	5.081827	-1.572247	1.846226
6	0.957036	2.458665	0.529941
1	0.853488	1.471413	0.977064
6	4.346679	-1.957737	-0.141461
6	4.336369	-0.501380	-0.547842
1	4.477868	-0.411115	-1.627740

1	5.174951	0.015217	-0.079140
6	3.965315	-4.305106	-0.652499
1	3.660012	-5.068136	-1.360226
6	3.044706	0.259158	-0.157556
1	2.874187	0.140089	0.915092
6	3.233537	1.773671	-0.431649
6	-4.847246	-1.489810	-1.110395
1	-4.617581	-0.799191	-1.915218
6	1.047626	-1.192337	-0.175439
6	-1.980649	1.854413	0.929560
1	-1.470062	2.201695	1.827303
6	-2.484545	-2.393298	-1.286291
1	-2.511650	-1.910670	-2.268336
1	-2.159240	-3.425220	-1.450349
6	4.372584	-4.665393	0.631326
1	4.384317	-5.708063	0.928577
6	1.584516	4.764431	0.877619
1	2.015829	5.469632	1.588488
1	0.876210	5.313183	0.254148
6	-1.894945	0.320280	0.959533
6	-2.483577	3.096089	-1.107622
1	-2.869370	2.379193	-1.840591
1	-2.074233	3.946586	-1.648353
6	-3.867220	-2.387709	-0.673100
6	-0.009556	-1.862588	-1.040426
1	0.239247	-2.925897	-1.101619
1	-0.001924	-1.467014	-2.060308
6	4.774557	-3.673969	1.528370
1	5.102215	-3.944524	2.526227
6	-4.193483	-3.279789	0.357198
1	-3.451259	-3.992335	0.703809
6	-6.426753	-2.364168	0.497914
1	-7.413158	-2.356542	0.947996
6	-6.117135	-1.475873	-0.531091
1	-6.863353	-0.773456	-0.886023
6	-1.409366	-1.704117	-0.418667
1	-1.405618	-2.143542	0.578190
6	-5.459789	-3.269011	0.939674
1	-5.693665	-3.969248	1.734117
6	-3.541734	3.462404	-0.067692
1	-4.534431	3.572408	-0.506172
1	-3.272144	4.406873	0.412004
6	-3.473032	2.306098	0.940909
1	-4.110409	1.481552	0.613762
1	-3.787887	2.591586	1.943776

Table S5. Coordinates (in Å) of the DFT optimized structure of P03 in aqueous solutions.

8	-2.049782	0.920529	-2.953036
1	-1.376027	-0.707136	0.832244
8	-1.129488	1.868354	2.435762

1	1.431034	-0.376658	1.206881
7	-1.233681	2.088729	-1.193351
7	0.825264	1.880846	1.304737
8	-1.013703	-2.234187	-1.908948
7	-1.519122	-0.882330	-0.151472
8	2.033240	0.432912	-1.781163
7	1.647974	-0.563651	0.238290
6	-0.527905	2.022770	1.369710
6	-0.864770	3.200737	-2.110145
1	-1.338920	3.038956	-3.073497
1	0.218338	3.215250	-2.265586
6	-4.498735	-1.485297	1.694877
1	-3.834378	-1.057449	2.438664
6	-1.076637	4.116451	0.089201
1	-1.699998	4.672064	0.789506
1	-0.033147	4.324641	0.337182
6	-5.447232	-1.476716	-0.516638
1	-5.524415	-1.042800	-1.508774
6	-1.364426	2.590031	0.197739
1	-2.389742	2.453948	0.528419
6	-4.568759	-0.915359	0.419410
6	-3.709266	0.277710	0.049417
1	-3.347377	0.753986	0.962244
1	-4.315417	1.017291	-0.481944
6	-5.280149	-2.593324	2.027353
1	-5.211994	-3.020863	3.021544
6	-2.529138	-0.114620	-0.869264
1	-2.915509	-0.778000	-1.641341
6	-1.920051	1.030129	-1.733860
6	4.881054	-1.546242	1.077726
1	4.449297	-1.278938	2.036721
6	-0.831501	-1.901671	-0.739305
6	1.684161	1.878906	0.109381
1	1.314924	2.572440	-0.640689
6	2.737742	-2.781680	0.516947
1	2.581055	-2.742543	1.599614
1	2.611229	-3.825952	0.216769
6	-6.146778	-3.146064	1.086003
1	-6.755903	-4.005444	1.342653
6	-1.348099	4.450376	-1.379320
1	-0.827492	5.351546	-1.706258
1	-2.418832	4.590243	-1.550848
6	1.786288	0.505254	-0.579345
6	1.639514	1.950316	2.539597
1	1.936226	0.952212	2.880456
1	1.053809	2.412420	3.330855
6	4.139634	-2.323608	0.181234
6	0.233713	-2.571652	0.110166
1	0.244963	-3.629411	-0.156806
1	0.011632	-2.496083	1.178747
6	-6.229709	-2.582067	-0.188398

1	-6.906920	-3.000314	-0.924849
6	4.724642	-2.666033	-1.045108
1	4.170649	-3.277533	-1.750685
6	6.739449	-1.461354	-0.466973
1	7.741907	-1.131571	-0.715741
6	6.170679	-1.117842	0.758811
1	6.730116	-0.519055	1.469201
6	1.627319	-1.963649	-0.179823
1	1.799059	-1.965797	-1.256886
6	6.011323	-2.238923	-1.369071
1	6.447419	-2.517116	-2.322243
6	2.847219	2.773200	2.087430
1	3.719514	2.608289	2.721101
1	2.595215	3.835768	2.122898
6	3.086094	2.324543	0.634885
1	3.781931	1.484093	0.604111
1	3.498505	3.117571	0.012169
